# A vaccine chatbot intervention for parents to improve HPV vaccination uptake among middle school girls: a cluster randomized trial

**DOI:** 10.1038/s41591-025-03618-6

**Published:** 2025-04-07

**Authors:** Zhiyuan Hou, Zhengdong Wu, Zhiqiang Qu, Liubing Gong, Hui Peng, Mark Jit, Heidi J. Larson, Joseph T. Wu, Leesa Lin

**Affiliations:** 1 School of Public Health, Fudan University, Shanghai, China; 2 National Health Commission Key Laboratory of Health Technology Assessment, Fudan University, Shanghai, China; 3 Laboratory of Data Discovery for Health Limited (D24H), Hong Kong SAR, China; 4 WHO Collaborating Centre for Infectious Disease Epidemiology and Control, School of Public Health, Li Ka Shing Faculty of Medicine, The University of Hong Kong, Hong Kong SAR, China; 5 Moonrise Initiative, Hong Kong SAR, China; 6 Chizhou Center for Disease Control and Prevention, Chizhou, China; 7 Jiading District Center for Disease Control and Prevention, Shanghai, China; 8 Department of Infectious Disease Epidemiology and Dynamics, London School of Hygiene & Tropical Medicine, London, UK; 9 Centre for the Evaluation of Vaccination, Vaccine & Infectious Disease Institute, University of Antwerp, Antwerp, Belgium; 10 Department of Health Metrics Sciences, University of Washington, Seattle, WA USA; 11 The University of Hong Kong-Shenzhen Hospital, Shenzhen, China; 12 Department of Global and Environmental Health, School of Global Public Health, New York University, New York, USA; 13 The Hong Kong Jockey Club Global Health Institute, Hong Kong SAR, China

**Keywords:** Public health, Health care

## Abstract

Conversational artificial intelligence, in the form of chatbots powered by large language models, offers a new approach to facilitating human-like interactions, yet its efficacy in enhancing vaccination uptake remains under-investigated. This study assesses the effectiveness of a vaccine chatbot in improving human papillomavirus (HPV) vaccination among female middle school students aged 12–15 years across diverse socioeconomic settings in China, where HPV vaccination is primarily paid out-of-pocket. A school-based cluster randomized trial was conducted from 18 January to 31 May 2024. The study included 2,671 parents from 180 middle school classes stratified by socioeconomic setting, school and grade level in Shanghai megacity, and urban and rural regions of Anhui Province. Participants were randomly assigned to either the intervention group (90 classes, 1,294 parents), which engaged with the chatbot for two weeks, or the control group (90 classes, 1,377 parents), which received usual care. The primary outcome was the receipt or scheduled appointment of the HPV vaccine for participants’ daughters. In intention-to-treat analyses, 7.1% of the intervention group met this outcome versus 1.8% of the control group (*P* < 0.001) over a two-week intervention period. In addition, there was a statistically significant increase in HPV vaccination-specific consultations with health professionals (49.1% versus 17.6%, *P* < 0.001), along with enhanced vaccine literacy (*P* < 0.001) and rumor discernment (*P* < 0.001) among participants using the chatbot. These findings indicate that the chatbot effectively increased vaccination and improved parental vaccine literacy, although further research is necessary to scale and sustain these gains. Clinical trial registration: NCT06227689.

## Abstract (web summary)

In a large trial involving 180 classes in different contexts in China, interactions with a chatbot provided to parents increased uptake and appointments for human papillomavirus vaccination, with stronger increases in rural areas.

## Main

Cervical cancer remains a major global health challenge, with 662,301 new cases and 348,874 deaths reported worldwide in 2022 (ref. ^1^). China faces a heavey disease burden, accounting for 22.8% of the global incidence and 16.0% of the deaths^2^. It is anticipated that China will face a continuing upward trend in these figures over the coming years^3,4^. Human papillomavirus (HPV) is responsible for almost all cervical cancer cases^5^. Vaccination against HPV has been shown to be a cost-effective measure to reduce cancer incidence, especially when it is a single dose and produced locally^6,7^. China has domestically produced two bivalent vaccines and effectively reduced its market price to US$48 per dose, while the imported nonavalent vaccine costs US$183 per dose. However, HPV vaccination is paid out-of-pocket in most parts of China, and its uptake remains very low. By 2022, only 10.15% of Chinese women aged 9–45 years had been vaccinated against HPV^8^. Coverage among female adolescents aged 9–14 years, who are recommended by the World Health Organization (WHO) to be vaccinated before sexual activity begins^9^, was less than 5% (refs. ^8,10^). This is markedly below the WHO’s 2030 target of 90% coverage by age 15 years^11^. To improve HPV vaccination coverage, a small but increasing number of Chinese cities or provinces have piloted government-funded vaccination programs for female middle school students since 2022.

Parental decision-making plays a crucial role in adolescent vaccination, requiring both parental consent and the minor’s assent. In traditional Chinese culture, conservative sexual norms create barriers to open discussions of sexual health^12^. As such, vaccine hesitancy among Chinese parents becomes a major barrier to HPV vaccination among their children, driven by low levels of knowledge about HPV and the vaccine, low confidence and willingness to accept vaccination^13,14^. A meta-analysis of studies from 2009 to 2023 revealed that, on average, only 61.0% of parents in mainland China were willing to vaccinate their children against HPV even if it was available for free^15^. Willingness to vaccinate decreases further when parents are required to pay for the vaccine outside standard healthcare packages^16–18^ and is notably lower in rural areas because of limited access to health information and economic constraints^15,19^. In addition, parental knowledge about HPV and its vaccine remains low^20,21^, even in the pilot regions where vaccines are publicly funded to support expanded HPV immunization for female middle school students^22,23^. To illustrate this knowledge gap, in Fujian province, one of the pilot regions, only 48.9% of mothers demonstrated a high level of knowledge about HPV^23^. In the Minhang district of Shanghai, parents hesitant about HPV vaccination for their daughters (aged 9–14 years) scored significantly lower on knowledge assessments, particularly among those not intending to vaccinate^24^. Moreover, concerns about vaccine safety exacerbate hesitancy, with more than 80% of parents wary of potential side effects^16^.

Conversational artificial intelligence (AI), often implemented using chatbots, uses large language models^25^ to enable new, automated, human-like interactions. Its emergence has the potential to both raise challenges and to overcome barriers in health communication and vaccine hesitancy^26,27^. These AI-driven platforms offer 24/7 access, allowing individuals to engage in discussions about vaccination at their convenience^28–30^. Studies across different vaccination contexts have demonstrated chatbots’ potential to enhance vaccine confidence and uptake^31–35^, with documented success in changing both attitudes and behavioral outcomes. However, two critical knowledge gaps remain. First, the mechanisms through which chatbot interventions influence an individual vaccination decision-making process remain poorly understood. Second, although chatbot interventions have shown promise in emergency vaccination campaigns such as COVID-19 (refs. ^36–38^), their effectiveness in contexts in which vaccines are not part of routine immunization programs and require substantial out-of-pocket costs requires investigation. Our study addresses these gaps by examining how a chatbot intervention might influence HPV vaccination in China, where substantial cost barriers exist, while also exploring the pathways through which such digital intervention can impact vaccine-related decision-making.

In this study, we conducted a cluster randomized trial to assess the effectiveness of a bespoke AI-powered vaccine chatbot in improving HPV vaccination among female middle school students in Shanghai and Anhui Province, China. The primary outcome was the receipt or scheduled appointment of the HPV vaccine. Secondary outcomes included HPV vaccination-specific consultations with health professionals, willingness to vaccinate, vaccine confidence, and vaccine literacy.

## Results

### Study participant disposition

**Fig. 1:**
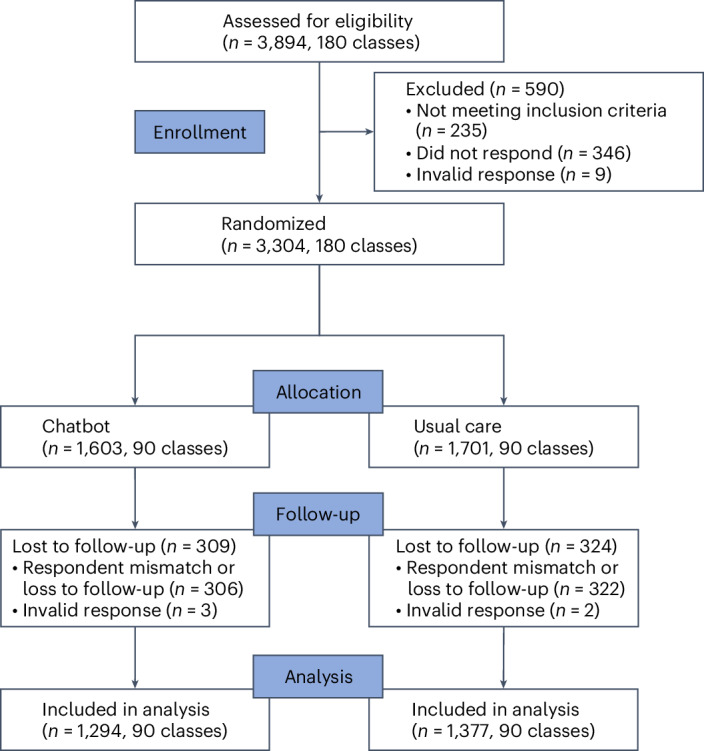
CONSORT flow diagram. The enrollment, randomization and follow-up of study participants from 180 classes across two study arms.

A school-based cluster randomized trial was conducted in Shanghai megacity, and urban and rural regions of Anhui Province from 18 January to 31 May 2024. A total of 180 classes were selected from 10 junior middle schools following the discussion with local education bureaus and schools, with 75 classes in megacity settings, 54 in urban areas and 51 in rural counties (Table 1). During the initial screening phase, 3,894 parents of female middle school students (grades 6–9, typically aged 12–15 years) were identified, and under the recruitment of class teachers, 3,304 parents agreed to participate in the trial and finished baseline survey (Fig. 1). Exclusions included 235 parents whose children had already received or were scheduled to receive the HPV vaccine, representing an overall vaccination rate of 6.62%, 346 parents owing to a nonresponse and 9 parents owing to an invalid response.

The 180 classes were randomly assigned in a 1:1 ratio to either the intervention group, which used the chatbot, or the control group, which received usual care, defined as local ongoing HPV vaccination promotion without additional interventions. Randomization was stratified by socioeconomic setting (megacity, urban or rural), school and grade. All parents of female school students were randomized by class into the intervention group (chatbot, *n* = 1,603) and control group (usual care, *n* = 1,701).

During the study period, 633 participants were lost to follow-up: 309 in the intervention group (306 because of respondent mismatch or loss to follow-up and 3 invalid responses) and 324 in the control group (322 because of respondent mismatch or loss to follow-up and 2 invalid responses). The final analysis included 2,671 parents: 1,294 in the intervention group (480 megacity, 432 urban and 382 rural settings) and 1,377 in the control group (487 megacity, 462 urban and 428 rural settings). The cluster numbers remained consistent throughout the study: 75 clusters in the megacity, 54 in urban areas and 51 in rural areas. Participants enrolled and not enrolled in the trial were comparable in most characteristics (Extended Data Table 1).

Table 2 presents the baseline characteristics of the enrolled participants in the intervention and control groups. Participating parents had a median age of 40 years (interquartile range (IQR) 37–43), while their daughters had a median age of 13 years (IQR 12–14). Mothers constituted 87.7% of participants, with 23.7% reporting that they had received HPV vaccination. The uptake of the HPV vaccine among mothers across socioeconomic settings was 31.5% in megacities, 20.7% in urban cities and 17.8% in rural settings. About 5.1% of the daughters were left-behind children, whose parents moved to another city for work but left them in their hometown to live with grandparents or guardians.

**Table 1:** Baseline characteristics of participating middle school classes

Classes	Total (*n* = 180)	Chatbot (*n* = 90)	Usual care (*n* = 90)
Grade, *n* (%)			
6	23 (12.8)	12 (13.3)	11 (12.2)
7	61 (38.9)	30 (33.3)	31 (34.4)
8	60 (33.3)	30 (33.3)	30 (33.3)
9	36 (20.0)	18 (20.0)	18 (20.0)
Region, *n* (%)			
Megacity	75 (41.6)	38 (42.2)	37 (41.1)
Urban	54 (30.0)	27 (30.0)	27 (30.0)
Rural	51 (28.3)	25 (27.8)	26 (38.9)

**Table 2:** Baseline characteristics of enrolled parents and their daughters

Characteristics	Total (*n* = 2,671; 180 classes)	Chatbot (*n* = 1,294; 90 classes)	Usual care (*n* = 1,377; 90 classes)
**Participants’ daughter**			
Grade, *n* (%)			
6	322 (12.1)	167 (12.9)	155 (11.3)
7	956 (35.8)	447 (34.5)	509 (37.0)
8	788 (29.5)	387 (29.9)	401 (29.1)
9	605 (22.7)	293 (22.6)	312 (22.7)
Age, years (mean ± s.d.)	13.1 ± 1.1	13.1 ± 1.1	13.1 ± 1.1
Only child, *n* (%)			
Yes	1,030 (38.6)	502 (38.8)	528 (38.3)
No	1,641 (61.4)	792 (61.2)	849 (61.7)
Left-behind child, *n* (%)			
Yes	137 (5.1)	67 (5.2)	70 (5.1)
No	2,534 (94.9)	1,227 (94.8)	1,307 (94.9)
Received sexual education, *n* (%)			
Yes	1,589 (59.5)	791 (61.1)	798 (58.0)
No	1,082 (40.5)	503 (38.9)	579 (42.0)
Influenza vaccinated within 2 years, *n* (%)			
Yes	814 (30.5)	376 (29.1)	438 (31.8)
No	1,857 (69.5)	918 (70.9)	939 (68.2)
**Participants**			
Region, *n* (%)			
Megacity	967 (36.2)	480 (37.1)	487 (35.4)
Urban	894 (33.5)	432 (33.4)	462 (33.6)
Rural	810 (30.3)	382 (29.5)	428 (31.1)
Parenthood, *n* (%)			
Mother	2,342 (87.7)	1,139 (88.0)	1,203 (87.4)
Father	329 (12.3)	155 (12.0)	174 (12.6)
Age, years (mean ± s.d.)	40.4 ± 4.6	40.3 ± 4.4)	40.5 ± 4.8
Education level, *n* (%)			
High education	1,167 (43.7)	587 (45.4)	580 (42.1)
Senior high school	482 (18.0)	226 (17.5)	256 (18.6)
Junior middle school and below	1,022 (38.3)	481 (37.2)	541 (39.3)
Formal employment, *n* (%)			
Yes	826 (30.9)	410 (31.7)	416 (30.2)
No	1,845 (69.1)	884 (68.3)	961 (69.8)
Household income, *n* (%)			
>300,000 CNY	258 (9.7)	122 (9.4)	136 (9.9)
200,000–300,000 CNY	316 (11.8)	163 (12.6)	153 (11.1)
1000,000–200,000 CNY	919 (34.4)	454 (35.1)	465 (33.8)
<100,000 CNY	1,178 (44.1)	555 (42.9)	623 (45.2)
Self or spouse HPV vaccinated, *n* (%)			
Yes	634 (23.7)	294 (22.7)	340 (24.7)
No	2,037 (76.3)	1,000 (77.3)	1,037 (75.3)

Study participants were enrolled from 180 middle school classes, with a chatbot group (*n* = 1,294) and a usual care group (*n* = 1,377). Baseline data were collected through standardized questionnaires. Left-behind children refers to children whose parents moved to another city for work. Education level indicates the highest level completed. Income categories are presented in Chinese Yuan, CNY (1 CNY ≈ 0.14 USD).

### Primary outcome

The primary outcome, measured by the receipt of or scheduled appointment for the HPV vaccine among female middle school students over a two-week intervention period, showed that 7.1% (92 out of 1,294) of parents in the chatbot group had scheduled or received an HPV vaccination for their daughters, compared with only 1.8% (25 out of 1,377) in the usual care group (Table 3). Among 1,294 participants in the chatbot group, 75 (5.8%) had scheduled and 17 (1.3%) had received an HPV vaccination, whereas among 1,377 participants in the usual care group, only 24 (1.7%) had scheduled and 1 (0.1%) had received an HPV vaccination. The intention-to-treat (ITT) analysis indicated a statistically significant increase in vaccine receipt or scheduled appointment after adjusting for confounding factors, showing that parents in the chatbot group were 3.85 times (adjusted relative risk: 3.85, 95% confidence interval (CI) 2.48–5.97) more likely to initiate HPV vaccination (either by scheduling or receiving the vaccine) than those in the usual care group (*P* < 0.001).

**Table 3:** Effect of HPV vaccine chatbot intervention on primary and secondary outcomes

		Pre-intervention	Post-intervention	Post–pre difference (95% CI)	Adjusted RR (95% CI)	Coefficient (95% CI)	*P* value
HPV vaccine receipt or scheduled appointment among female students, *n* (%)	Chatbot	–	92 (7.1)	7.1 (5.7 to 8.5)	3.85 (2.48 to 5.97)		<0.001
	Usual care	–	25 (1.8)	1.8 (1.1 to 2.5)			
HPV vaccination-specific consultation, *n* (%)	Chatbot	–	635 (49.1)	49.1 (46.3 to 51.8)	2.73 (2.41 to 3.09)		<0.001
	Usual care	–	242 (17.6)	17.6 (15.6 to 19.7)			
Parental willingness to vaccinate their daughter, *n* (%)^a^	Chatbot	961 (74.3)	917 (70.9)	−3.4 (−6.8 to 0.0)	1.02 (0.98 to 1.07)		0.325
	Usual care	993 (72.1)	937 (68.1)	−4.1 (−7.5 to −0.7)			
High vaccine confidence, *n* (%)^b^	Chatbot	794 (61.4)	860 (66.5)	5.1 (1.4 to 8.8)	1.02 (0.97 to 1.08)		0.375
	Usual care	796 (57.8)	830 (60.3)	2.5 (−1.2 to 6.1)			
HPV-related literacy, mean (s.d.)	Chatbot	6.3 (3.1)	7.1 (2.8)	0.7 (0.5 to 1.0)		0.70 (0.52 to 0.88)	<0.001
	Usual care	6.1 (3.2)	6.2 (3.1)	<0.1 (−0.2 to 0.3)			
HPV-related literacy—knowledge, mean (s.d.)	Chatbot	3.9 (2.0)	4.3 (1.9)	0.5 (0.31 to 0.61)		0.38 (0.26 to 0.50)	<0.001
	Usual care	3.7 (2.0)	3.8 (2.0)	0.1 (−0.1 to 0.2)			
HPV-related literacy—rumor screening, mean (s.d.)	Chatbot	2.5 (1.5)	2.7 (1.4)	0.3 (0.2 to 0.4)		0.32 (0.21 to 0.43)	<0.001
	Usual care	2.4 (1.5)	2.4 (1.5)	<−0.1 (−0.2 to 0.1)			

The analysis included 2,671 participants (1,294 in the chatbot group, 1,377 in the usual care group). Post–pre difference indicates the change from pre-intervention to post-intervention. GEEs were used for categorical outcomes and mixed-effects models for continuous outcomes, with class as the cluster unit and adjusting for stratification variables and baseline characteristics. Adjusted relative risks (RR) and coefficients represent the intervention effects assessed through interaction terms between intervention group and time, with *P* value as the statistical significance.

^a^Parental willingness was defined as parents who were ‘strongly willing’ or ‘willing’ to vaccinate their daughter against HPV. Parents who reported in the follow-up survey that they had scheduled an appointment or their daughter had been vaccinated were also categorized as ‘strongly willing’.

^b^Parents who ‘strongly agree’ or ‘agree’ with statements regarding the importance, efficacy, and safety of HPV vaccine, demonstrate high vaccine confidence.

### Secondary outcomes

The secondary outcomes included HPV vaccination-specific consultation, parental willingness to vaccinate their daughter against HPV, HPV vaccine confidence and HPV-related literacy scores. The chatbot intervention significantly increased the proportion of participants consulting health professionals about getting their daughter vaccinated against HPV in the two weeks of this trial (Table 3). Post-intervention, a marked difference was observed between the two groups, with 49.1% of parents in the chatbot group consulting health professionals compared with 17.6% in the usual care group. This resulted in an adjusted relative increase, indicating that parents in the chatbot group were 2.73 times (95% CI 2.41–3.09) more likely to consult health professionals about HPV vaccination for their daughters than parents in the usual care group (*P* < 0.001), thereby demonstrating the chatbot’s effectiveness in encouraging parents to seek professional advice on HPV vaccination.

Changes in parents’ willingness to vaccinate their daughters against HPV showed a slight decrease, from 74.3% to 70.9% in the chatbot group and from 72.1% to 68.1% in the usual care group, with the post–pre differences between the two groups not statistically significant (*P* = 0.325). By contrast, high vaccine confidence saw a slight increase in both groups, from 61.4% to 66.5% in the chatbot group and from 57.8% to 60.3% in the usual care group, with increases of 5.1% and 2.5% respectively. However, these post–pre increases also did not show a statistically significant difference between the two groups (*P* = 0.375).

Parents’ HPV-related literacy in the chatbot group showed a statistically significant improvement, with the mean score increasing from 6.3 to 7.1 of a total of 10 points, compared with a minimal change in the usual care group, which increased from 6.1 to 6.2. The chatbot group exhibited an average improvement of 0.70 points (95% CI 0.52–0.88) in HPV literacy compared with the usual care group. For knowledge assessment (of 6 points), the chatbot group demonstrated a statistically significant improvement in knowledge, increasing their scores from 3.9 to 4.3, whereas the usual care group showed minimal change from 3.7 to 3.8, with statistical significance in two groups (*P* < 0.001). For rumor identification (of 4 points), the chatbot group showed a notable improvement from 2.5 to 2.7, whereas the usual care group remained at 2.4, with statistical significance in two groups (*P* < 0.001). Extended Data Table 2 presents the comparison for each of the ten literacy-related statements.

### Subgroup analysis

**Fig. 2:**
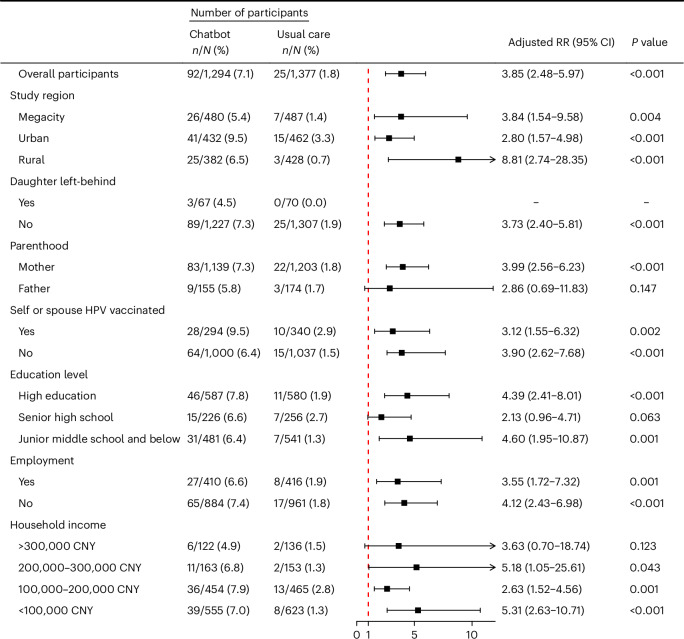
Stratified GEE to compare HPV vaccine receipt or scheduled appointment between two arms. Forest plot shows adjusted RR with two-sided 95% CI comparing HPV vaccine receipt or scheduled appointment between chatbot and usual care groups in each subgroup. The data present estimates from ITT analyses using stratified GEE models, with class as the cluster unit. The models adjusted for stratification variables and confounders including parents’ characteristics (age, education level, employment, income, self or spouse HPV vaccination) and daughters’ characteristics (age, only child, left-behind child, sexual education, influenza vaccination). No estimate was provided for the ‘Daughter left-behind—Yes’ subgroup owing to zero events in the usual care group. Statistical significance was set at *P* < 0.05 (two-tailed). CNY, Chinese Yuan.

**Extended Data Fig. 1:**
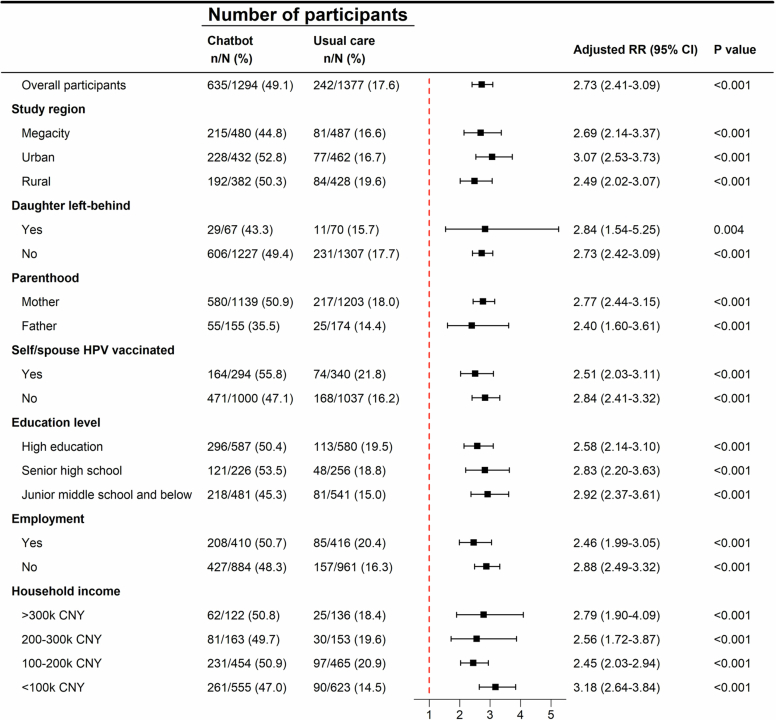
Stratified GEE to compare HPV vaccination-specific consultation with health professionals between two arms. Forest plot shows adjusted RR with two-sided 95% CI comparing HPV vaccination-specific consultation rates between chatbot and usual care groups in each subgroup. The data present estimates from ITT analyses using stratified GEE models, with class as the cluster unit. The models adjusted for stratification variables and confounders including parents’ characteristics (age, education level, employment, income, self/spouse HPV vaccination) and daughters’ characteristics (age, only child, left-behind child, sexual education, influenza vaccination). Statistical significance was set at P < 0.05 (two-tailed). CNY, Chinese Yuan.

**Extended Data Fig. 2:**
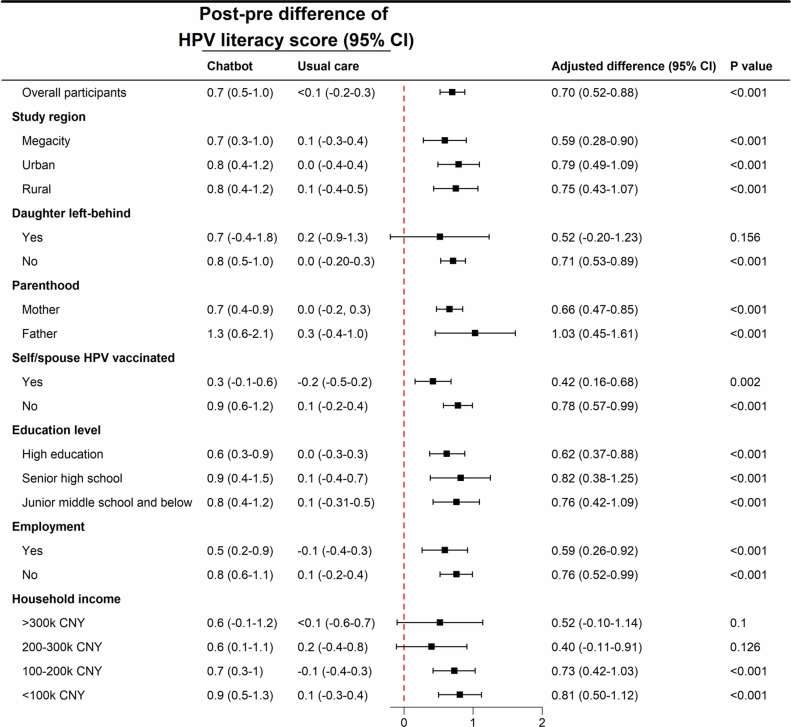
Stratified mixed-effects model to compare HPV literacy between two arms. Forest plot shows adjusted mean differences with two-sided 95% CI comparing post-pre changes in HPV literacy scores between chatbot and usual care groups in each subgroup. The data present estimates from ITT analyses using stratified mixed-effects models, with class as the cluster unit. The models adjusted for stratification variables and confounders including parents’ characteristics (age, education level, employment, income, self/spouse HPV vaccination) and daughters’ characteristics (age, only child, left-behind child, sexual education, influenza vaccination). Statistical significance was set at P < 0.05 (two-tailed). CNY, Chinese Yuan.

The subgroup analysis revealed that the chatbot intervention significantly enhanced HPV vaccine receipt or scheduled appointment, consultation and literacy in nearly all demographic subgroups and regions compared with usual care (Fig. 2 and Extended Data Figs. 1 and 2). Notably, in rural areas, vaccine receipt or scheduled appointment in the chatbot group was 8.81 times higher (95% CI 2.74–28.35) than that in the usual care group. When mothers used the chatbot, vaccine receipt or scheduled appointment for their daughters was 3.99 times higher (95% CI 2.56–6.23) in the chatbot group compared with the usual care group.

### Chatbot engagement

In this trial, 1,054 of 1,294 participants (81%) in the intervention group were confirmed to have used the HPV vaccine chatbot based on our chatbot data matching, but 3 of these users did not engage in any substantive dialog. The remaining 19% may not have been matched because of nonuse of the chatbot, errors in information provided for matching or use by another family member. The chatbot users and nonusers in the intervention group were comparable in most characteristics (Extended Data Table 3).

Engagement with the chatbot was similar across different regions, with 80% (386 out of 480) in megacities, 84% (362 out of 432) in urban areas and 79% (303 out of 382) in rural areas. Participants typically logged into the chatbot once (IQR 1–2). The median duration of each user session was 2.9 min (IQR 1.4–5.6 min). Participants asked a median of 12 questions (IQR 10–20), with rural participants asking a median of 13 questions (IQR 9–24) and a maximum of 139 questions.

For the analysis of user interactions with the chatbot, all 16,950 user-posed questions were manually categorized by two public health experts to classify them according to their primary concerns. Leading topics included vaccine safety and side effects, discussed by 931 users (88.6%) in 4,031 questions (23.8%), and vaccination age and eligibility, addressed by 724 users (68.9%) in 2,239 questions (13.2%). In addition, there was also high interest in the specifics of different HPV vaccine types and the overall necessity and benefits of vaccination. Practical concerns such as the cost of vaccines, scheduling for vaccination and vaccine availability were frequently raised, alongside inquiries into the effectiveness of the vaccines and proper vaccination procedures. For a detailed breakdown of the topics discussed and the frequency of each category, see Extended Data Table 4.

Table 4 further reveals the associations between chatbot engagement characteristics and vaccination outcomes. Overall, parents with high engagement levels in the chatbot intervention were 2.16 times (95% CI: 1.35–3.44) more likely to initiate HPV vaccination for their daughters than those with low engagement. Among the four engagement indicators, those with high interaction frequency were 2.06 times (95% CI: 1.28–3.31) more likely to initiate HPV vaccination than those with low interaction frequency, whereas usage duration, number of questions asked and diversity of topics discussed did not show statistically significant associations with vaccination outcomes. In addition, users who engaged with the nurse persona were 2.08 times (95% CI: 1.25–3.45) more likely to initiate HPV vaccination than those who exclusively interacted with the vaccine expert persona.

**Table 4:** Association between chatbot engagement characteristics and HPV vaccine receipt or scheduled appointment in the intervention group (*n* = 1,051)

Chatbot engagement	Primary outcome, *n/N (%)*	Adjusted RR (95% CI)	*P* value
Overall engagement			
High	21/180 (11.7)	2.16 (1.35-3.44)	0.002
Low	48/871 (5.5)		
Interaction frequency			
>2	43/467 (9.2)	2.06 (1.28-3.31)	0.003
1-2	26/584 (4.5)		
Usage duration			
>9.2 min	23/263 (8.7)	1.55 (0.97-2.47)	0.077
≤9.2 min	46/788 (5.8)		
No. of questions			
>10	50/794 (6.3)	0.90 (0.54-1.49)	0.680
≤10	19/257 (7.4)		
Topic diversity			
≥6	45/656 (6.9)	1.14 (0.70-1.83)	0.604
<6	24/395 (6.1)		
Chatbot persona			
Nurse or combined	32/333 (9.6)	2.08 (1.25-3.45)	0.005
Expert only	37/718 (5.2)		

Adjusted RR with 95% CI were estimated using log-binomial models adjusted for clustering effects and parents’ and daughters’ characteristics. Overall engagement was defined as having high engagement across all four indicators: interaction frequency (>2 interactions, 75th percentile); usage duration (>9.2 min, 75th percentile); number of questions asked (>10); and diversity of topics discussed (≥6 topics, median). Expert only group includes participants who interacted with expert persona only, while nurse or combined group includes participants who used nurse persona, regardless of their expert persona use.

### Per-protocol analysis

A per-protocol (PP) analysis (Extended Data Table 5) among the 1,051 parents who actively engaged with the chatbot showed consistent intervention effects, supporting the robustness of our findings. In terms of the primary outcome, the adjusted analysis indicated a substantial increase in the chatbot group’s likelihood of scheduling or receiving an HPV vaccination for their daughters compared with the usual care group. Specifically, parents in the chatbot group were 3.42 times (95% CI 2.19–5.35) more likely to initiate vaccination (*P* < 0.001). Further analysis on health professional consultations showed a similar pattern, with parents in the chatbot group being 2.81 times (95% CI 2.48–3.18) more likely to seek professional advice on HPV vaccination in the two weeks of this trial compared to the usual care group (*P* < 0.001).

Although the effects of the chatbot intervention on the willingness to vaccinate and HPV vaccine confidence were not statistically significant, it did have a significant effect on HPV-related literacy. The chatbot group demonstrated a marked improvement of 0.73 points (95% CI 0.54–0.92) in HPV-related literacy scores compared to the usual care group (*P* < 0.001).

## Discussion

This study presents an HPV vaccine chatbot intervention in China through a cluster randomized trial, aimed at increasing HPV vaccination among female middle school students. It found that the chatbot intervention significantly increased vaccination in this demographic, enhanced parental engagement in vaccination consultations and improved HPV-related literacy. These findings are consistent with existing literature suggesting that digital interventions, such as chatbots, client reminders and provider education, can effectively increase HPV vaccine uptake^33–35,39^. Parents in the intervention group were more proactive in seeking advice from health professionals, suggesting that chatbots can effectively promote engagement with healthcare services and encourage positive health behavior^40^. In addition, the intervention enhanced parents’ ability to discern factual information from misinformation, thereby improving HPV-related literacy and supporting vaccine confidence^41,42^. Our findings further indicate that higher engagement levels with the chatbot were significantly associated with greater HPV vaccine receipt or scheduled appointment, particularly among parents with higher interaction frequency. The nurse persona was more effective in promoting vaccination than the expert persona, indicating chatbot’s effectiveness by the use of simpler and more conversational language. These results demonstrate a promising pathway for positive behavioral change toward vaccination and underscore the scalability potential of chatbots in health promotion.

It is important to note that the chatbot prompted half of parents to consult health professionals about vaccinating their daughters, but only 7.1% then scheduled or received HPV vaccination for their daughters. In our study, HPV vaccination was paid out-of-pocket, and the nonavalent vaccine preferred by participants was in short supply^43^. Its high cost and the long waiting period may be key barriers to converting consultations into actual vaccination behavior. Since 2022, an increasing number of cities or provinces in China have introduced local government-funded HPV vaccination programs to address the cost barriers. This trial used a two-week intervention to strike a balance between assessing the impact of chatbot use and minimizing the confounding factors. However, parents may not have had enough time to schedule an appointment and take their daughters to receive the HPV vaccination, which may partly contribute to the low vaccination rate in the intervention group. In addition, the chatbot did not affect parents’ willingness and confidence to vaccinate their daughters. The limited impact on deeper, long-term attitudes may be attributed to the limited duration of the intervention^44^. Deeply ingrained personal beliefs and societal norms likely exerted considerable influence and were difficult to reverse with a short-term intervention^45–47^. This underscores the need for further research to explore the effects of prolonged digital interventions on parental attitudes toward vaccination.

Moreover, this study highlights the chatbot’s efficacy in improving HPV vaccination practices across almost all demographic subgroups and regions. Despite some rural parents reporting login difficulties, we observed encouraging improvements in vaccine receipt or scheduled appointment across both rural and nonrural areas. AI technologies could potentiate discriminatory bias and health disparities or instead, foster access to healthcare for underserved populations to mitigate health inequities^48–50^. Our study indicates the positive role of an AI-powered chatbot in addressing critical disparities in healthcare access and preventive measures.

The strengths of this study are multifaceted. It explores the impact of an AI-powered HPV vaccine chatbot on vaccination coverage, using a cluster randomized trial design that added scientific rigor to the evaluation. The interactive nature of the chatbot effectively bridged gaps in public knowledge and motivation by providing personalized and timely information. Furthermore, our study assessed the effects of the chatbot, demonstrating its immediate influence on vaccination. In addition, a number of individuals who were not recruited to the study accessed the chatbot, and although these external users were excluded from the final analysis, their participation underscores broader public interest and suggests potential for scalability. This successful implementation suggests its potential as a valuable tool for healthcare providers in managing vaccination and broader health applications, demonstrating the innovative application of AI technology in public health interventions.

The study has several limitations. First, HPV vaccination requires out-of-pocket payment and the preferred nonavalent vaccine is in short supply in our settings—these factors may undermine the chatbot’s capacity to effectively boost vaccination rates. Our results may not apply to settings in which HPV vaccination is publicly funded and routinely given in schools. Further research is needed to investigate the effectiveness of chatbots for publicly funded vaccination, particularly as publicly funded HPV vaccination becomes more widespread in China and around the world. Second, we did not directly communicate with the daughters themselves, which may have impacted their autonomy in making vaccination decisions. Third, participants in the control group were required to complete the baseline survey which may itself have stimulated their interest in the HPV vaccine. It was observed that in the control group, 17.6% of parents had consulted health professionals about HPV vaccination, and 1.8% had scheduled or received an HPV vaccination for their daughters over a two-week study period. This could potentially lead to an underestimation of the chatbot intervention’s effects. Conversely, the regular reminders to parents in the intervention arm about the chatbot may have kept HPV vaccination in their minds and by itself may have increased interest in the topic. Fourth, although the study was designed for single-parent participation, the online surveys and participation could not ensure that the same parent completed the baseline and follow-up surveys. If one parent interacted with the intervention’s chatbot and another parent filled out the questionnaires, it could also introduce bias and compromise the reliability of the data. Fifth, because of the small number of schools in the study, classes allocated to control and intervention arms may have been in the same school. This may have caused some cross-contamination if children in the intervention classes talked to friends in the control classes about HPV vaccination, causing further underestimation of the chatbot’s effectiveness. Lastly, although we anticipated considerable hindrances due to rural network infrastructure, our findings revealed that the chatbot was relatively accessible across different regions. However, some rural parents did report connectivity issues, suggesting that individual connectivity challenges could influence chatbot usage. This finding underscores the need for further exploration into specific factors affecting chatbot adoption and effectiveness across various geographical settings.

In conclusion, this study demonstrates the efficacy of a chatbot intervention in improving HPV vaccination, parental HPV-related literacy and healthcare consultation rates regarding HPV and its vaccine in China. The chatbot effectively enhanced engagement with health services and improved knowledge and rumor discernment, crucial for dispelling misinformation. However, to sustain and amplify these achievements, additional strategies to increase user engagement and optimize chatbot design are imperative. The findings suggest that AI chatbots represent a promising intervention for improving HPV vaccine uptake among targeted populations. This specific application demonstrates the potential utility of digital health tools in enhancing access to preventive healthcare services. Although these results are encouraging, they primarily pertain to this singular intervention. Further research is necessary to explore the scalability and effectiveness of AI chatbots across different vaccines and other public health interventions, which could potentially address broader social disparities in healthcare access.

## Methods

### Trial design and setting

This study was a cluster randomized trial approved by both the Institutional Review Board (IRB) of Fudan University School of Public Health and the Human Research Ethics Committee of the University of Hong Kong. It was conducted in middle schools with class as the unit of randomization. Schools were chosen from three representative regions in China, including the metropolitan area of Shanghai Jiading, the urban setting of Guichi in Anhui Province and the rural counties of Dongzhi and Qingyang, also in Anhui Province. These areas were not covered under China’s ‘Action Plan for the Accelerated Elimination of Cervical Cancer (2023–2030)’^12^, which provides government-funded vaccination for female middle school students in participating locations. Therefore, the study areas required that HPV vaccinations be paid out-of-pocket without government subsidies. It was registered on ClinicalTrials.gov (NCT06227689). The study protocol is presented in the Supplementary Information.

### Participants

Participants were enrolled if they met the following inclusion criteria: (1) participants were parents of female students currently enrolled in participating middle schools (grades 6–9, where students typically range from 12 to 15 years of age); (2) the female HPV vaccine-eligible child of the surveyed parent had not received an HPV vaccine, did not have an HPV vaccination appointment scheduled and did not have any contraindications to receiving the HPV vaccine; (3) participants were free of mental health disorders or visual or reading disabilities that could prevent their full participation in and completion of the intervention activities; and (4) participants provided informed consent and expressed a willingness to actively participate throughout the study. Exclusion criteria were defined as individuals not meeting the aforementioned inclusion criteria. All participants provided informed consent to participate this trial.

### Sampling, randomization and masking

In each of three regions, three or four middle schools were selected based on the economic development and geographical location, resulting in a total of 180 classes from grades 6–9 in ten schools. One parent (either the father or mother) of all eligible female school students from the selected classes was invited to participate in the trial, with approximately 20 parents per class.

The classes as clusters were randomly assigned to either the intervention or control groups in a 1:1 ratio, using a stratified randomization approach. Stratification was based on three factors: three regions (megacity, urban and rural settings), ten schools and four grade levels (grades 6–9) to ensure even distribution and minimize potential confounders. Using class lists in each grade in a school, computer-generated randomization was used to determine whether class 1 and class 2 were assigned to intervention or control group, and classes with odd numbers (classes 3 and 5) followed the assignment of class 1, and classes with even numbers (classes 4 and 6) followed the assignment of class 2. This procedure was repeated independently across all grade levels of all schools. The randomization process was blinded to schools, teachers and participants. The nature of the intervention did not allow for masking of the intervention to class teachers, participants or study implementers.

### Intervention

In this study, we developed an AI-powered chatbot tailored for HPV vaccine consultation, designed specifically for the Chinese context. The foundation of this chatbot is an expansive knowledge database, constructed with information sourced exclusively from healthcare authorities such as the Chinese Center for Disease Control and Prevention and the National Health Commission of China. Each piece of information has been thoroughly verified by public health experts. This database integrates data on the HPV infection burden, susceptibility and severity, along with in-depth details about the HPV vaccine, including its importance, efficacy, safety and recommended demographics and timing for vaccination. It also covers types and costs of vaccines, societal norms such as vaccination guidelines, expert recommendations and vaccination trends both in China and internationally. In addition, the database includes misinformation and fact-checking contents to address vaccine-related misinformation and provides information about vaccination services such as locations and appointment scheduling. The chatbot operates with two roles: an ‘expert’ and a ‘nurse’, both of which rely on the knowledge base to ensure accurate information delivery. The ‘expert’ role provides responses that are highly professional and detailed, including references for users to verify the information. By contrast, the ‘nurse’ role offers responses that are warm and empathetic, designed to make users feel more comfortable during interactions.

To differentiate from standard large language models like ChatGPT, which may provide extensive but not always context-specific or up-to-date information, our chatbot uses advanced linguistic technologies through GPT-4 (ref. ^51^) coupled with retrieval-augmented generation and prompt engineering^52^. This integration allows the chatbot to dynamically pull accurate and relevant information directly from our specialized database in real-time, rather than relying solely on pre-trained knowledge. The chatbot underwent a two-week testing phase with stakeholders, including six public health professionals from local Centers for Disease Control and Prevention (CDCs) and 20 parents of vaccine-eligible female adolescents. Based on their feedback, several improvements were implemented: refinements to the chatbot’s role design, enhanced user interface design and the addition of preset frequently asked questions to facilitate user interaction. These modifications enhanced the chatbot’s functionality and relevance in practical scenarios.

**Extended Data Fig. 3:**
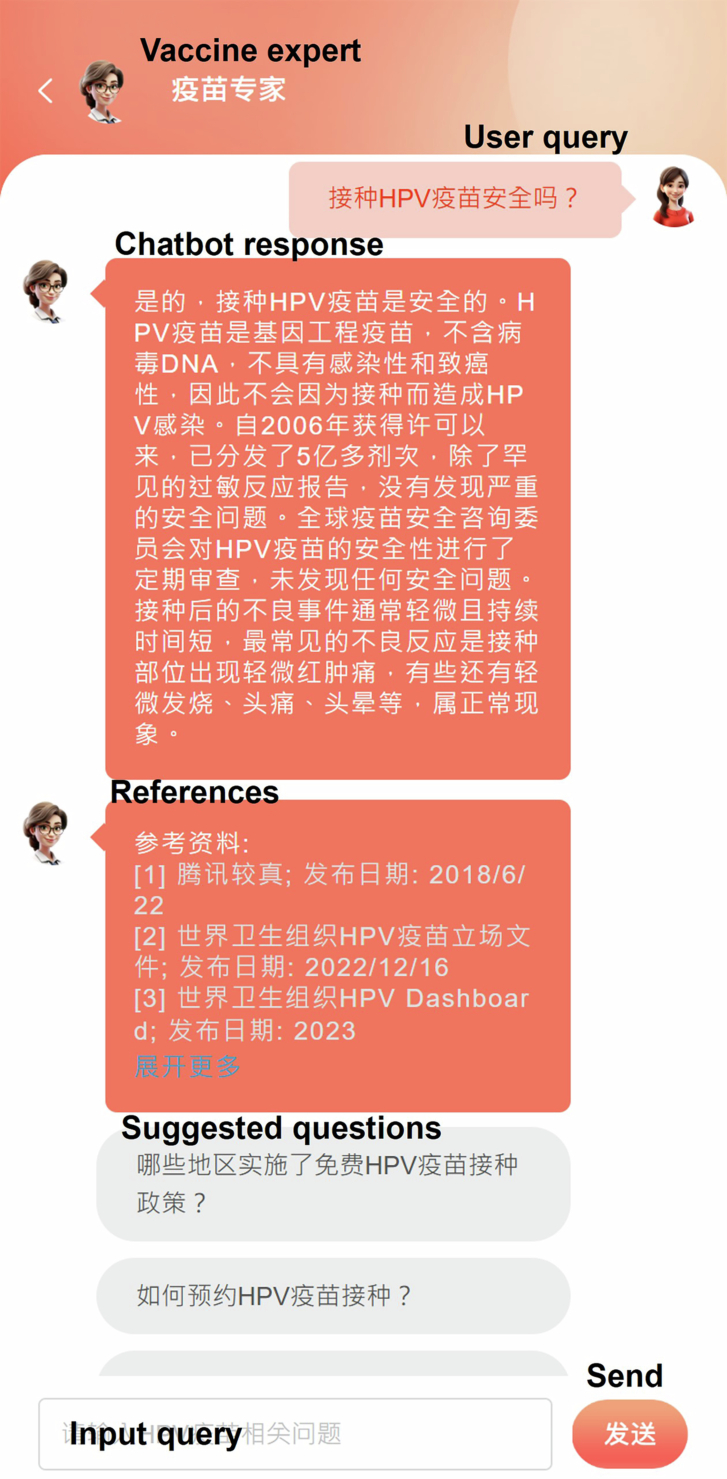
User interface for Chinese HPV vaccine chatbot. This figure shows a vaccine expert-user interaction in the HPV vaccine chatbot. The interface displays a user’s query about HPV vaccine safety, followed by the expert’s evidence-based response with scientific references. Key interface components include the expert’s avatar, user query box, response panel with citations, and suggested follow-up questions.

This tailored design leverages evidence-based and personalized responses to ensure the chatbot meets users’ needs for reliable and contextually relevant information about HPV vaccination. The AI-powered chatbot is accessible via WeChat and through web browsers at https://hpvchatbot.social-insight.ai. The user interface of the chatbot is depicted in Extended Data Fig. 3. More details about the development and evaluation of this chatbot will be reported in a separate manuscript.

The chatbot was designed to improve vaccine literacy and uptake by addressing public concerns and reducing hesitancy toward HPV vaccination. Individuals with questions or concerns about HPV vaccination were hypothesized to engage with the chatbot when it was initially introduced. Also, since 2022, an increasing number of regions had introduced or were considering introduction of free HPV vaccination for female middle school students under 15 years old. Following discussions with local stakeholders, a short (two-week) intervention period was used for this trial to avoid potential contextual interference.

During the two-week intervention, participants in the intervention group were informed that the chatbot was available for use at their convenience, with coordinators sending the chatbot link every four days to remind them to use it. Conversely, the control group received usual care, referring to standard local HPV vaccination promotion practices that did not include additional interventions, and had no access to the chatbot during the trial period. To meet ethical requirements, they were provided access to the chatbot after the study concluded.

### Study procedures

This study included both baseline and follow-up surveys with two-week chatbot intervention. A pilot study began on 18 January 2024, with formal participant enrollment commencing on 20 February 2024 following protocol approval. School-level consent for this study was established by providing written information about the study and obtaining a signed letter of affirmation from each school’s principal. Parental consent was secured through a multifaceted approach. Printed letters containing the participant information and consent form were given to students by class teachers, who then delivered these letters to their parents or guardians. Each letter provided detailed study information and included a QR code for easy access to the survey. In addition, class teachers promoted the study’s informed consent information through digital parent communities, such as WeChat groups or DingTalk, enhancing accessibility and convenience for the parents’ review. At the initiation of the electronic questionnaire, a summarized version of the informed consent was presented to reinforce comprehension and uphold informed consent principles. Participants provided informed consent to participate this trial during their screening or baseline visits. The informed consent included nine key elements: (1) study purpose, (2) procedures, (3) risks, (4) benefits, (5) compensation details, (6) confidentiality measures, (7) right to withdraw, (8) signature for informed consent, and (9) investigator’s statement. Parents were afforded a 14-day period to opt out of the study, providing them the autonomy to withdraw at any stage. Participant information and consent forms were thoroughly reviewed by researchers during the baseline assessment to verify that consent had been fully informed and properly obtained from the parents of female middle school students.

Before the intervention began, an initial assessment was conducted for both the intervention and control groups. This assessment collected baseline data on participants and their daughters’ sociodemographics, parental HPV vaccination history, flu vaccination history of daughters, HPV vaccine information exposure and seeking, health professional consultation about HPV vaccine and HPV vaccine-related knowledge, attitudes, willingness and behaviors.

After the two-week intervention period, a follow-up assessment was conducted. This follow-up assessment included both the intervention and control groups, covering evaluations of demographics, primary outcomes and secondary outcomes. In addition, participants in the intervention group underwent an acceptability and feasibility assessment of the HPV vaccine chatbot. The HPV vaccination status of female middle school students was recorded during the follow-up assessment. Both baseline and follow-up questionnaires are provided in the Supplementary Information.

### Outcomes

The primary outcome was the receipt or scheduled appointment of the HPV vaccine, determined by whether the participants’ daughters were vaccinated or had actively scheduled a vaccination in the two-week intervention period. Receipt of an HPV vaccine was verified using official vaccination records, whereas scheduled vaccine appointment was measured in the post-intervention survey because of the absence of a unified appointment system. Both vaccine receipt and scheduled appointment were included as outcome measures because of the HPV vaccine supply situation in China. There is strong preference but heavy shortage of supply for the nonavalent HPV vaccine, although the bivalent, quadrivalent and nonavalent vaccines are available in China^10^. A long waiting period such as half a year or even longer is usually needed for nonavalent HPV vaccination.

Secondary outcomes included: (1) whether participants had consulted health professionals about getting their daughters vaccinated against HPV in the two-week post-intervention period; (2) parental willingness to vaccinate their daughters against HPV; (3) HPV vaccination literacy, measured by whether parents correctly answered ten statements relating to HPV or its vaccine, including six knowledge-related statements and four rumor-related statements, and rumor-related statements indicate parental ability to discern misinformation from accurate information (Extended Data Table 2); and (4) HPV vaccine confidence, measured using the Vaccine Confidence Index^53^, which assesses perceptions of the vaccine’s importance, effectiveness and safety. In addition, chatbot usage outcomes were measured by participant engagement rates, frequency of interactions, duration of use and main topics of inquiry regarding the HPV vaccine, to evaluate the chatbot’s effectiveness in enhancing informed health decisions.

To ensure alignment with real-world implementation conditions, the outcome measures were modified during the development of the trial protocol. As a pragmatic trial, extensive engagement with local CDCs and relevant stakeholders was undertaken to refine and finalize the protocol between September 2023 and February 2024. In the original protocol, HPV vaccine receipt or scheduled appointment was designated as the primary outcome, while parental willingness to vaccinate was co-listed as a primary outcome, reflecting early uncertainties regarding access to linked vaccination records in the registration system. Following formal engagement with local CDCs and a pilot study, contextual and mechanistic insights facilitated access to vaccination records, allowing vaccine receipt or scheduled appointment to remain the primary outcome. Consequently, willingness to vaccinate was reclassified as a secondary outcome in the final protocol, recognizing its role as an intermediate mechanism rather than a definitive measure of real-world vaccine uptake. Further, study site stakeholders recommended adding HPV vaccination-specific consultation with health professionals as a secondary outcome, given the two-week intervention period. This addition helped to unpack the intervention’s impact mechanism, because engagement with healthcare providers may mediate the chatbot’s influence on vaccination decisions. These refinements, informed by realist principles, were integrated into the final protocol to guide the trial’s implementation, ensuring the trial remained both rigorous and contextually responsive (Supplementary Information). The trial registration was later updated accordingly to reflect these modifications.

### Sample size

The sample size was calculated based on detecting a meaningful difference in the primary outcome of HPV vaccination among female middle school students, comparing its differences before and after the intervention. According to national estimates of the China CDC, first-dose HPV vaccine coverage among female adolescents aged 9–14 years in China is around 5% (refs. ^8,10^). We hypothesized that an effective intervention could increase this rate by five percentage points, reaching 10%. According to the literature on school-based cluster randomized trials^54,55^, the intracluster correlation coefficient (ICC) was assumed to be 0.05.

In China, the average class size includes approximately 20 female students. Considering students who had either already received the vaccination or had scheduled it, as well as parents who might decline survey participation, we anticipated that around 10–15 parents (*m*) per class would be eligible participants. The design effect was calculated by 1 + (*m* − 1) × ICC, which yielded a range of 1.45 to 1.70. For this trial, we adopted a cluster design effect of 1.5 (*m* = 11). Assuming a power of 80%, a two-sided significance level of 0.05 and incorporating this cluster design effect, we determined that a sample size of 648 participants per arm would be sufficient to detect a change of this magnitude.

To account for potential loss to follow-up (estimated at 20–30%), the sample size per arm was then expanded to 900, for a total of 1,800 participants. Consequently, a total of 162 classes were required for the study. These classes were randomized to intervention and control groups in a 1:1 ratio, stratified by city, school and grade levels. The stratification resulted in approximately 81 classes in each group. The study aimed to recruit approximately 1,800 parents, with 900 in the intervention group and 900 in the control group, ensuring that the sample size requirements were met.

1 $${m}_{1}=\frac{{\left({Z}_{1-\alpha /2}+{Z}_{1-\beta }\right)}^{2}\left[{P}_{1}\left(1-{P}_{1}\right)+{P}_{2}(1-{P}_{2})\right]}{{\Delta }^{2}}\times {\mathrm{DE}}$$

2 $${m}_{2}=\frac{{m}_{1}}{(1-d)}$$

where sample size (*m*_1_ and *m*_2_) represents the number of participants before or after considering the dropout rate (*d*). *P*_1_ and *P*_2_ denote the vaccination rates among female middle school students in the intervention and control groups, respectively. Δ signifies the clinically important difference in treatment proportions, and DE represents the cluster design effect.

In this trial, the ICC was estimated to be 0.113 for the primary outcome of HPV vaccination among female middle school students. The power analysis showed that this trial had a power of 98.7% to detect the difference in the primary outcome between intervention and control groups.

### Statistical analysis

Both ITT and PP analyses were conducted to evaluate the intervention effects. The intervention effect was assessed through the interaction term between intervention group and time. The PP analysis was conducted among parents whose data were successfully matched with the chatbot’s usage records to verify the robustness of our findings.

Mixed-effects models and generalized estimating equations (GEE) were used to evaluate the effectiveness of the chatbot intervention on primary and secondary outcomes, adjusting for potential confounders. Mixed-effects models were used for continuous variables (HPV-related literacy) to handle multilevel data structure, and GEE was applied for categorical outcomes (HPV vaccine receipt or scheduled appointment, HPV vaccination-specific consultation with health professionals, willingness to vaccinate and vaccine confidence). All models accounted for the cluster design with class as the cluster unit and controlled for stratification variables. The models also adjusted for parents’ characteristics (parenthood, age, education level, employment, income and self or spouse HPV vaccination) and their daughters’ characteristics (age, only child, left-behind child, sexual education and influenza vaccination), with stepwise reduction of covariates used to ensure model convergence when needed.

In addition, we used log-binomial generalized linear models to assess the associations between chatbot engagement characteristics and vaccine receipt or scheduled appointment in the intervention group. The models adjusted for the same confounders as described above, including clustering effects and parents’ and daughters’ characteristics. High overall engagement was operationally defined as achieving high levels across all four metrics (interaction frequency, usage duration, number of questions asked and topic diversity). Interaction frequency and usage duration, both asymmetrically distributed, were dichotomized at their 75th percentiles (two interactions and 9.2 min, respectively). The number of questions asked was dichotomized at ten questions, corresponding to the chatbot’s reminder protocol, and topic diversity, following a normal distribution, was categorized at the median (six or more categories). We also compared vaccine receipt or scheduled appointment between users who exclusively interacted with the expert persona and those who engaged with the nurse persona. Given that the majority of users interacted with the expert persona, any interaction with the nurse persona was used to define the nurse persona group.

Statistical analyses were performed using STATA v.15.1 and R v.4.4.1 statistical software. Two-tailed tests with a significance level of 0.05 were used.

### Inclusion and ethics statement

This study was conducted in alignment with evidence-based clinical practice guidelines and received approval from the IRB of Fudan University School of Public Health and Human Research Ethics Committee of the University of Hong Kong. The original version of the protocol was approved by the IRB of Fudan University School of Public Health on 19 September 2023. Following discussions with local CDCs and feedback from relevant stakeholders, it was revised in January 2024 and finalized and approved in February 2024. The study holds IRB approval number IRB#2023-09-1082.

A reporting mechanism was implemented to ensure participant safety throughout the study. Participants could report any concerns directly to the study coordinator using the established protocol at any point during the research. These reports would be reviewed by the principal investigator, ensuring that all concerns were addressed promptly and documented according to the IRB at Fudan University School of Public Health and the Human Research Ethics Committee guidelines at the University of Hong Kong.

### Safety and adverse events

Adverse events were monitored through an established reporting protocol. No adverse events were reported during the study.

### Reporting summary

Further information on research design is available in the Nature Portfolio Reporting Summary linked to this article.

## Online content

Any methods, additional references, Nature Portfolio reporting summaries, source data, extended data, supplementary information, acknowledgements, peer review information; details of author contributions and competing interests; and statements of data and code availability are available at 10.1038/s41591-025-03618-6.

## Supplementary information

Supplementary Information Supplementary Document 1. Study protocol for HPV vaccine chatbot (translated version). Supplementary Document 2. Study protocol for HPV vaccine chatbot (Chinese version). Supplementary Document 3. Survey questionnaires. Supplementary Document 4. CONSORT checklist.

Reporting Summary

## Figures and Tables

**Extended Data Table 1:**
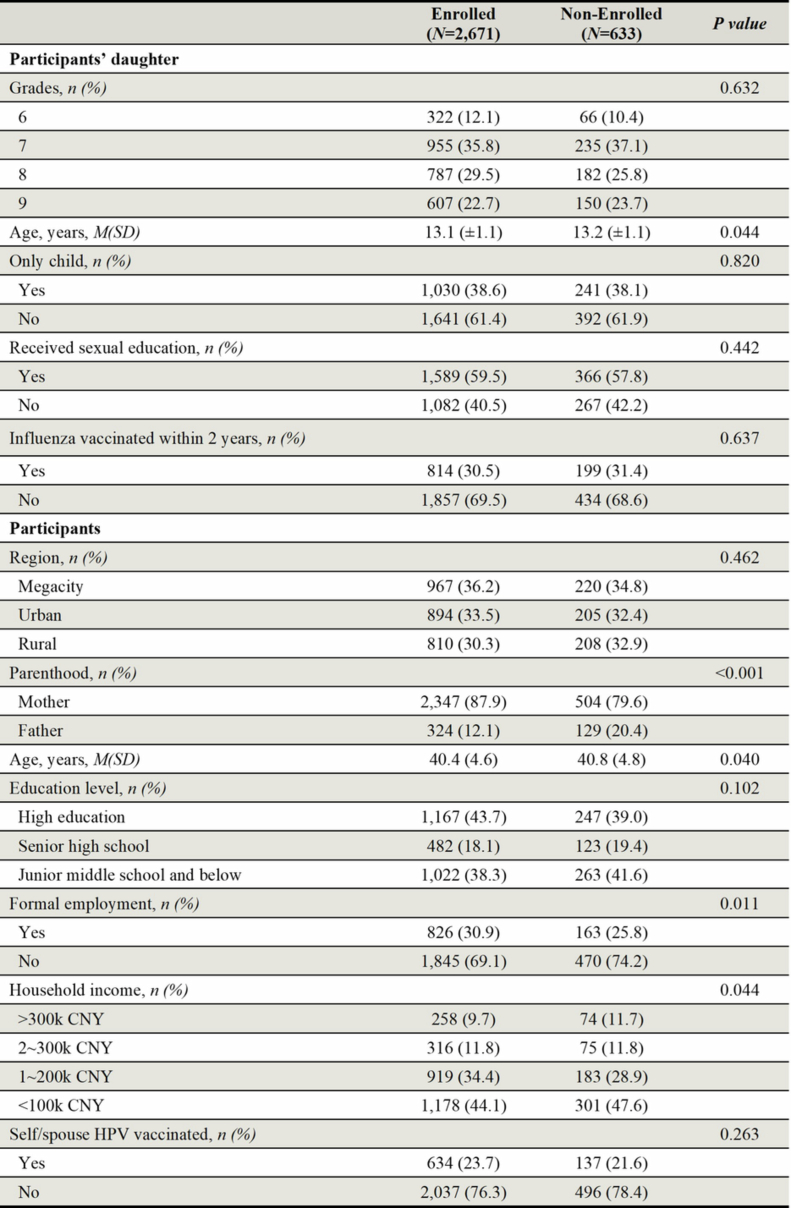
Comparative characteristics of enrolled and non-enrolled participants

Data are shown as n (%) or mean (s.d.). Study enrolled 2,671 participants and 633 were non-enrolled. P values were calculated using chi-square test for categorical variables and t-test for continuous variables. CNY, Chinese Yuan (1 CNY ≈ 0.14 USD).

**Extended Data Table 2:**
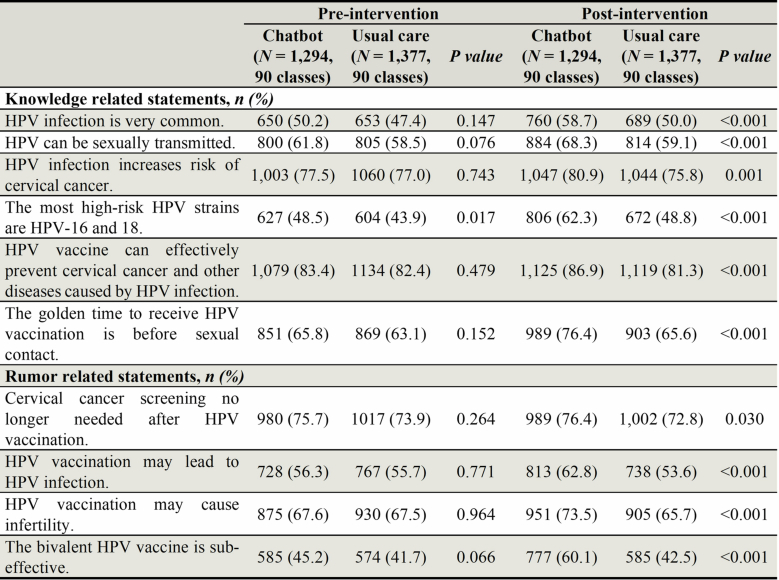
Proportions of parents correctly answered HPV or its vaccine related statements

Data are shown as n (%). The table presents the proportion of correct responses to each statement before and after the intervention in both groups. Statements were categorized into knowledge (6 items) and rumor (4 items) related components. P values were calculated using chi-square test to compare parental statement between chatbot and usual care groups.

**Extended Data Table 3:**
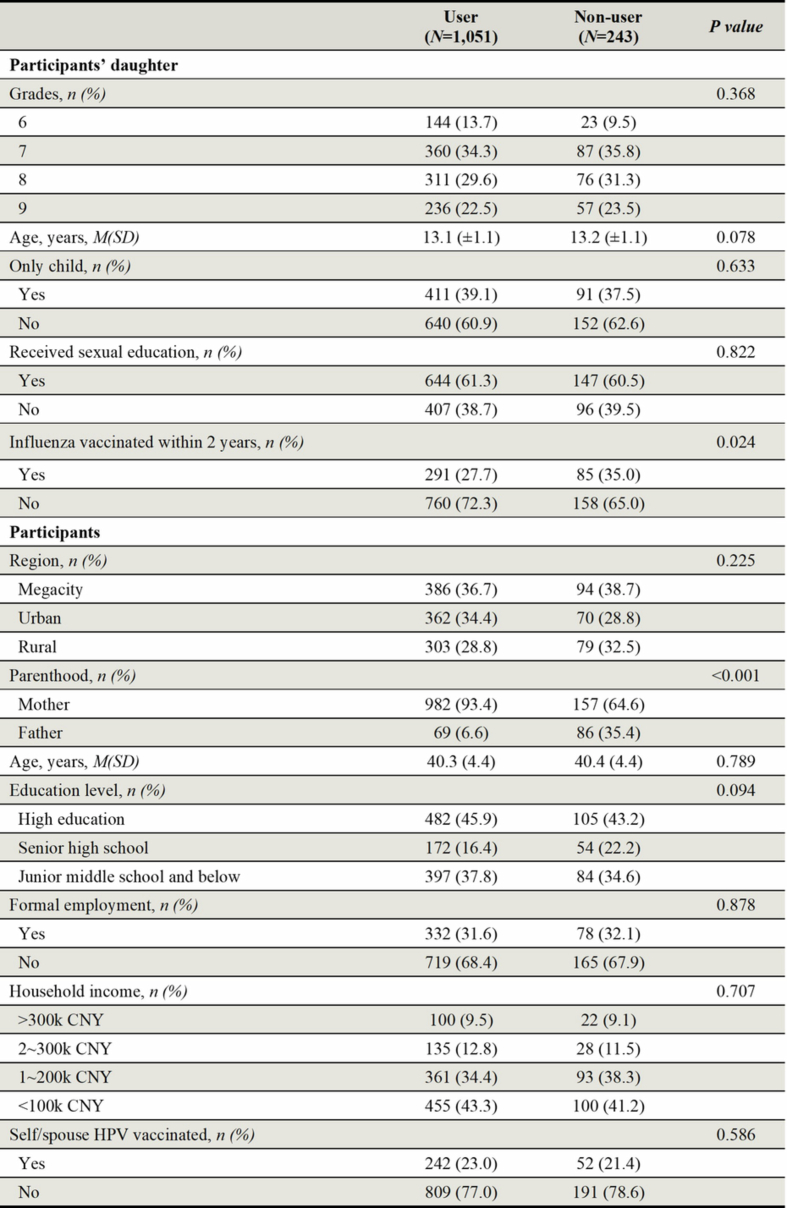
Comparative characteristics of chatbot users and non-users in the intervention group

Data are shown as n (%) or mean (s.d.). Among 1,294 participants in the intervention group, 1,054 were confirmed chatbot users and 240 were non-users based on chatbot data matching. P values were calculated using chi-square test for categorical variables and t-test for continuous variables. CNY, Chinese Yuan (1 CNY ≈ 0.14 USD).

**Extended Data Table 4:**
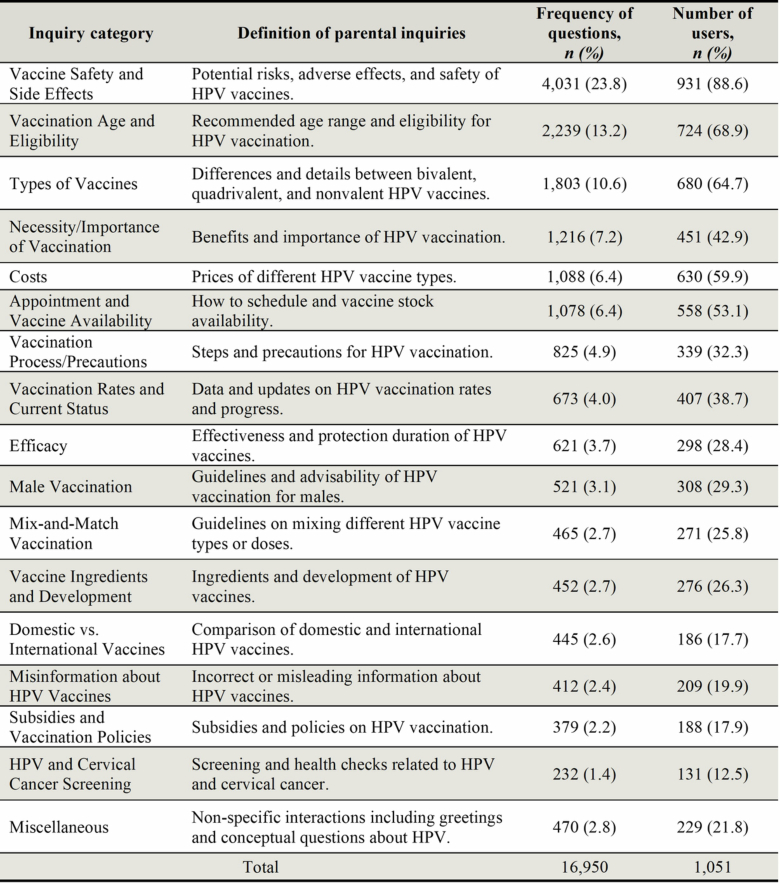
Distribution of parental inquiries to the HPV vaccine chatbot in the intervention group

Data are shown as n (%). The analysis included 16,950 questions from 1,051 users who accessed the chatbot among 1,294 participants in the intervention group. Questions were independently categorized by their primary topic by two experts, with individual users having inquiries across multiple categories.

**Extended Data Table 5:**
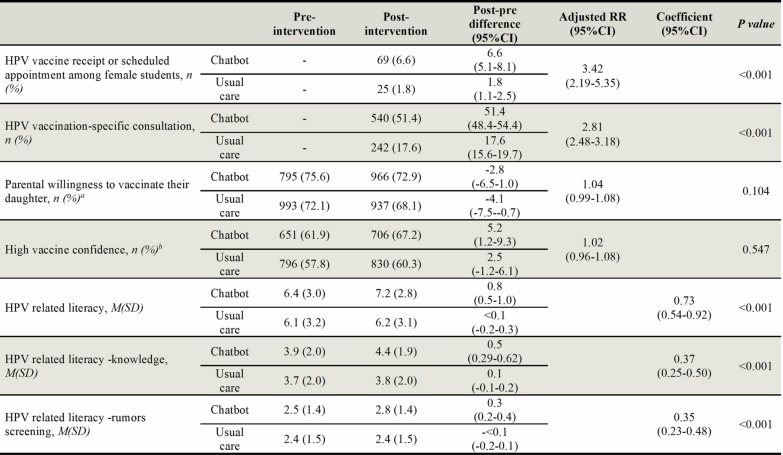
Per-protocol analysis of HPV vaccine chatbot intervention effects on primary and secondary outcomes

Data are shown as n (%) or mean (s.d.). The analysis included 1,051 participants whose data were successfully matched with the chatbot usage records and 1,377 participants in the usual care group. Post-pre difference indicates the change from pre-intervention to post-intervention. GEEs were used for categorical outcomes and mixed-effects models for continuous outcomes, with class as the cluster unit and adjusting for stratification variables and baseline characteristics. Adjusted RR and coefficients represent the intervention effects assessed through interaction terms between intervention group and time, with P value as the statistical significance.

^a^Parental willingness was defined as parents who were ‘strongly willing’ or ‘willing’ to vaccinate their daughter against HPV. Parents who reported in the follow-up survey that they had scheduled an appointment or their daughter had been vaccinated were also categorized as ‘strongly willing’.

^b^Parents who ‘strongly agree’ or ‘agree’ with statements regarding the importance, efficacy, and safety of HPV vaccine, demonstrate high vaccine confidence.

## Data Availability

The data are not publicly available, and making the data publicly available would require additional consent due to the need to protect participant privacy and confidentiality. Researchers interested in accessing the data should submit requests to the corresponding author (L. Lin, leesa.lin@lshtm.ac.uk or Z. Hou, zyhou@fudan.edu.cn), explaining the analyses planned. Access to data will be provided upon application, with a timeline of one month determined in accordance with the request. All approved users must sign a data use agreement that specifies confidentiality requirements, restricts data usage to the approved analyses, and prohibits any attempt to identify study participants. Source data are provided with this paper.

## Code availability

All codes are freely available on GitHub at https://github.com/wu-zhengdong/HPV-vaccine-chatbot.git.
