# Supplementary material for: A vaccine chatbot intervention for parents to improve HPV vaccination uptake among middle school girls: a cluster randomized trial
Source: Nat Med. 2025 Apr 7;31(6):1855–62. doi: 10.1038/s41591-025-03618-6 (PMC12176647; doi:10.1038/s41591-025-03618-6)
Supplement: Supplementary file 1 — Supplementary Document 1. Study protocol for HPV vaccine chatbot (translated version). Supplementary Document 2. Study protocol for HPV vaccine chatbot (Chinese version). Supplementary Document 3. Survey questionnaires. Supplementary Document 4. CONSORT checklist. [file 41591_2025_3618_MOESM1_ESM.pdf]

nature medicine

Supplementary information https://doi.org/10.1038/s41591-025-03618-6

A vaccine chatbot intervention for parents to improve HPV vaccination uptake among middle school girls: a cluster randomized trial

In the format provided by the authors and unedited

Supplementary information

Appendix index

Supplementary Document 1: Study protocol for HPV vaccine chatbot (translation version)

Supplementary Document 2: Study protocol for HPV vaccine chatbot (Chinese version)

Supplementary Document 3: Survey questionnaires

Supplementary Document 4: CONSORT Checklist

复旦大学公共卫生学院
School of Public Health, Fudan University
138 Yi Xue Yuan Road
Shanghai 200032, China

Supplementary Document 1: Study protocol for HPV vaccine chatbot (translation version)

Development of a chatbot intervention for HPV vaccine and evaluation of its impact on vaccine confidence in China

(Translation from Chinese final version February 2024)

School of Public health, Fudan University

Laboratory of Data Discovery for Health Limited, Hong Kong Science Park

School of Public Health, The University of Hong Kong

February 18, 2024

Abstract: Human papillomavirus (HPV) vaccination is highly cost-effective in preventing cervical cancer. China, being the world's second-highest burden of cervical cancer, has alarmingly low HPV vaccination rates, particularly among adolescents, the priority population for HPV vaccination. A key barrier is the lack of awareness and understanding of the HPV vaccine among adolescents' parents. The limited effectiveness of traditional interventions underscores the need for innovative strategies to improve parental knowledge and vaccination uptake for adolescents. This study utilizes artificial intelligence (AI) technology to develop an HPV vaccine chatbot and implements chatbot interventions among parents of adolescents. A cluster randomized controlled trial (cRCT) will assess its impact on parents' HPV vaccine knowledge, vaccine confidence, willingness to vaccinate their daughters, and their daughters' HPV vaccination uptake. Additionally, the study will assess the acceptability, scalability, and sustainability of the chatbot intervention through implementation science evaluation, to enhance its value in the public health practice. The HPV vaccine chatbot has the potential to support both vaccine communication and intervention.

I. Research Background

1. Research significance

Cervical cancer is a significant global health threat, and HPV vaccination is recognized as one of the most cost-effective preventive measures. The World Health Organization (WHO) recommends integrating the HPV vaccine into national immunization programs (NIP), prioritizing vaccination for girls aged 9-14. However, China, the country with the second-highest burden of cervical cancer globally, faces alarmingly low HPV vaccination rates, particularly among adolescents. Research indicated that limited awareness and understanding of the HPV vaccine among parents of adolescents are the major obstacles. Traditional interventions, such as face-to-face education and community outreach, have proven effective, but their inherent limitations in reach and scalability highlight the need for innovative solutions. Chatbots, which demonstrated their utility during the COVID-19 pandemic for health communication, offer a new approach to improving public awareness of HPV vaccines. However, their effectiveness in promoting HPV vaccine acceptance and their potential for large-scale implementation require systematic evaluation.

1.1 Low HPV vaccination rates in China, especially among adolescents, who are the priority group for protection

HPV vaccination is effective in preventing cervical cancer, with existing evidence indicating that earlier vaccination yields superior preventive outcomes. The WHO advocates for incorporating the HPV vaccine into national immunization programs (NIP), prioritizing vaccination for girls aged 9-14 years, and in 2020, proposed the "Global Strategy to Accelerate the Elimination of Cervical Cancer". China has also joined this strategy and, in January 2023, issued the "Cervical Cancer Elimination Action Plan (2023-2030)," which aims to pilot, continuously promote, and support HPV vaccination services for eligible girls over the next

decade by exploring innovative pathways. However, China has not yet included the HPV vaccine in the NIP, and prior to this, HPV vaccination in China primarily focused on female adults and university students, with insufficient attention given to adolescent girls, who represent the most cost-effective group. It is estimated that from 2018 to 2020, the cumulative HPV vaccination rate among eligible women aged 9-45 years in China was only 2.24%, and the rate among adolescents was less than 2%.

1.2 Limited awareness of cervical cancer and HPV vaccines among parents and adolescents remains a major barrier to vaccination uptake

The barriers to adolescents HPV vaccination include inadequate vaccine supply, high costs, and a lack of public awareness. Currently, bivalent, quadrivalent, and nine-valent HPV vaccines have been approved in China, and an increasing number of regions have started offering free vaccination to adolescent girls, addressing supply and cost issues. However, some barriers continue to hinder HPV vaccination among adolescents, such as limited awareness of HPV vaccines, poor understanding of cervical cancer risks, and insufficient knowledge about vaccination benefits among adolescents and their parents. The misinformation and rumors about HPV vaccines on the internet can also mislead both adolescents and their parents. Additionally, the nine-valent HPV vaccine was only approved for ages 16-26 in China until September 2022. Some people have delayed vaccination, preferring to wait for the nine-valent HPV vaccine rather than considering other available vaccine types.

1.3 Chatbots that emerged during the COVID-19 pandemic provide an alternative approach to improving public knowledge about HPV vaccination. Their effectiveness and implementation value warrant systematic evaluation

Traditional interventions to improve vaccination knowledge rely on health education programs, professional lectures, and healthcare provider recommendations. These approaches involve one-way information delivery, where recipients passively receive content. This unidirectional communication fails to meet specific informational needs of target populations. In China 's healthcare system, strained doctor-patient relationships have created a challenging environment for vaccine recommendations. This context has particularly affected healthcare providers' willingness to recommend Category 2 vaccines (non-mandatory, self-paid vaccines), including HPV vaccines. Furthermore, traditional interventions approaches face practical constraints: they require substantial resources, involve lengthy implementation periods, and remain difficult to scale and maintain over time.

During the COVID-19 pandemic, artificial intelligence (AI)-powered chatbots emerged as a novel approach in public health communication. These chatbots deliver demand-driven, interactive interventions to address public needs for health information, differing from traditional passive approaches. The chatbots can reach users through social media and online platforms, offering efficient and scalable implementation. These characteristics suggest their potential application in HPV vaccination programs. They can provide targeted information and support vaccination decisions, serving as tools to promote vaccine uptake. These applications

warrant the development of an HPV vaccine chatbot to evaluate its impact on vaccine-related knowledge and potential value in promoting vaccination.

Research questions:

1) How can an HPV vaccine chatbot be developed to deliver scientifically accurate and appropriate responses? What is the feasibility of utilizing the chatbot as an intervention tool to improve public confidence in HPV vaccination?

2) How effective is the chatbot interventions on improving HPV vaccine-related knowledge, awareness, confidence among parents, and HPV vaccine uptake of their daughters? What factors influence the effectiveness of the chatbot intervention?

3) How acceptable is the vaccine chatbot to key stakeholders? How can we systematically evaluate the implementation and scalability of this chatbot intervention?

2. Research Innovations

This study presents three innovations:

1) Develops an artificial intelligence (AI)-powered chatbot through interdisciplinary research in artificial intelligence and public health to promote HPV vaccination and enhance vaccine communication effectiveness..
2) Evaluates the chatbot through dialogue quality assessment, feasibility assessment as an intervention tool, and a clinical trial to assess its impact on HPV vaccination.
3) Uses implementation science methods to evaluate the chatbot intervention's acceptability, applicability, and scalability in real-world settings.

3. Literature Review

3.1 Awareness of cervical cancer and HPV vaccination among adolescents and their parents in China

Since introducing the HPV vaccine to China in 2016, five types of HPV vaccines have been approved for use by 2022. These include three imported vaccines (GlaxoSmithKline's (GSK) bivalent vaccine, Merck's quadrivalent and nine-valent vaccines) and two domestic bivalent vaccines. Despite the HPV vaccine being available in China for six years, the overall vaccination rate remains low, with only 2.24% of eligible women vaccinated. The vaccination rate for girls aged 9-14, the recommended target age group for HPV vaccination, is below 2%.

1) Awareness of cervical cancer and HPV vaccination among adolescents

Low HPV vaccination rates among adolescents are attributed to inadequate vaccine awareness, supply shortages, and high costs. Key issues include low perceived risk of cervical cancer, insufficient knowledge about HPV and its vaccine, and concerns about vaccine safety. A 2016 national survey revealed that only 32.8% of adolescents were aware of cervical cancer, 70% did not perceive themselves at risk of cervical cancer, and only 12.9% had heard of HPV and 17.1% had heard of the HPV vaccine. By 2020, the HPV vaccine awareness among

adolescents reached 65%. However, this increase was primarily observed among university students (79%), with students aged 10-14 still demonstrating the lowest levels of awareness (23%) and vaccination intent (43%). Lack of knowledge about the HPV vaccine was the main reason (76%) for reluctance to get vaccinated.

2) Awareness of cervical cancer and HPV vaccination among parents of adolescents

Parental awareness and acceptance of the HPV vaccine influence adolescent vaccination rates. However, the level of knowledge among Chinese parents remains insufficient. A meta-analysis covering 19 provinces in China found that the average awareness rate of the HPV vaccine among the Chinese people was only 16%, with approximately 60% of parents expressing willingness to vaccinate their children. Another meta-analysis reported that awareness of HPV and its vaccine among parents of adolescents was 28% and 19%, respectively, with only 41% willing to vaccinate their children. Moreover, misinformation and rumors have affected public understanding of the HPV vaccine. This limited understanding has led some parents to pursue more expensive, higher-valency vaccines, delay vaccinating their children, causing them to miss the optimal vaccination age.

3.2 Current status and trends of HPV vaccine awareness and uptake interventions

1) Status quo of traditional interventions

Traditional vaccination interventions, such as health education, promotional lectures, and healthcare provider recommendations, have demonstrated effectiveness in enhancing adolescents' awareness and willingness to receive the HPV vaccine. However, these approaches have several limitations. First, participants typically receive information passively, limiting their engagement. Furthermore, these interventions are resource-intensive and time-consuming. The short-term nature of these interventions also presents challenges for scalability and sustainability. To achieve widespread HPV vaccination coverage, alternative intervention strategies that overcome these limitations are needed.

2) Trends in novel interventions: Chatbot technology

During COVID-19 pandemic, research on vaccine-related chatbots emerged. Chatbots for COVID-19 vaccines provided information to support vaccination decision-making through: (1) disease-related knowledge, such as susceptibility and severity; (2) vaccine-related information, including the importance, safety, and efficacy of vaccines; (3) eligibility for vaccination; (4) current vaccination status of the population; (5) how to schedule a vaccination appointment; (6) vaccination advice, such as pre-and post-vaccination precautions; and (7) combating misinformation and negative news. A study conducted in Hong Kong at the end of 2021 found that chatbots could effectively reduce COVID-19 vaccine hesitancy, increase vaccine confidence, and promote vaccination uptake. In the population hesitant or unwilling to be vaccinated, chatbot interventions reduced vaccine hesitancy by 14.7%, while willingness to receive the COVID-19 vaccine and booster shots increased by 30% and 47.4%, respectively. Studies from Hong Kong and the United States both reported over 70% public acceptance of

chatbots, particularly in combating misinformation and negative media coverage. Several vaccine chatbots have already been launched internationally, including VIRA by Johns Hopkins University in the U.S., Corowa-kun developed by Japanese researchers, and ChatSure from Thailand's Ministry of Public Health. Notably, in November 2022, OpenAI released its chatbot, ChatGPT, which sparked widespread discussion and research within both industry and academia due to its advanced conversational capabilities. The adaptability and scalability of chatbots render them a viable tool for widespread implementation. Compared to traditional interventions, chatbot interventions offer irreplaceable advantages, including reduced costs and enhanced sustainability, delivering considerable value for application in the vaccination domain.

3.3 Research GAPs in HPV vaccine interventions and chatbot application in China

Currently, research on HPV vaccination in China primarily focuses on evaluating vaccination rates and intentions, with limited empirical intervention studies. To date, there are no studies exploring chatbot application for vaccination promotion. In contrast, chatbots have gained significant popularity internationally. During the COVID-19 pandemic, researchers integrated chatbots into social media platforms such as WhatsApp, Facebook, and Line to support pandemic control efforts, including promoting COVID-19 vaccination, assessing individual risks, and monitoring health symptoms. The widespread use of social media and the internet facilitates chatbot accessibility and usability. In China, social media platforms like WeChat and Douyin (TikTok) are widely used, creating potential opportunities for the joint development of chatbots. However, research on the application of chatbots to promote vaccination in China remains limited. Further studies are needed to assess the effectiveness of chatbots within the Chinese context and to evaluate their potential application value in the vaccination promotion.

II. Research Scope

1. Research Objective

This study aims to develop an artificial intelligence (AI)-powered chatbot to promote HPV vaccination and assess its effectiveness as an intervention in delivering HPV vaccine information. A cluster randomized controlled trial (cRCT) will be conducted to evaluate the chatbot's impact on parents' HPV vaccine knowledge, vaccine confidence, willingness to vaccinate their daughters, and their daughters' HPV vaccination uptake. The study will further explore the chatbot's acceptability and scalability to enhance its public health impact.

2. Research Design

1) Development of the HPV vaccine chatbot as an intervention tool

Guided by the BeSD (Behavioral and Social Drivers of vaccination) framework, we will design a chatbot question-and-answer (Q&A) database focusing on HPV vaccine-related topics. The initial development will utilize ChatGPT, a state-of-the-art large language model (LLM). We will evaluate its ability to respond to HPV vaccine-related queries in terms of

accuracy and scientific rigor. Any insufficient or inaccurate responses will be refined to create a more reliable HPV vaccine chatbot. Following completion of the trial, we will create an independent HPV vaccine chatbot using a generative pre-trained model developed in China.

2) Implementing the chatbot intervention and evaluating its impact on parents' HPV vaccine knowledge, vaccine confidence, willingness to vaccinate their daughters, and their daughters' HPV vaccination uptake

We will implement the HPV vaccine chatbot across three socioeconomically diverse locations: Shanghai Megacity, Guichi District (an urban area) and two rural counties (Dongzhi and Qingyang) of Chizhou City in Anhui Province. We will conduct a cluster randomized controlled trial with pre- and post-intervention surveys among parents of middle school girls. This design will evaluate the chatbot's impact on parents' HPV vaccine knowledge, vaccine confidence, willingness to vaccinate their daughters, and their daughters' HPV vaccination uptake, providing evidence for its effectiveness in promoting HPV vaccination.

3) Evaluating the acceptability and scalability of the chatbot intervention

We will conduct qualitative interviews to examine both user and professional perspectives on the chatbot. Users will be interviewed about their acceptance, experiences, preferences, and suggestions, while healthcare professionals will provide recommendations for improvement and dissemination. Based on this feedback, we will optimize the chatbot. We will then conduct implementation research to evaluate the intervention's scalability and explore its potential expansion to other vaccination programs, such as influenza vaccination.

III. Research Methodology and Technical Framework

1. Developing HPV vaccine knowledge base for chatbots: A Behavioural and Social Drivers of Vaccination approach

The HPV vaccine knowledge base for the chatbot will be developed based on the Behavioural and Social Drivers of Vaccination (BeSD) theory, which outlines the behavioural and social factors influencing vaccination. This model, introduced by the WHO in 2022, categorizes these drivers into four key modules:

1) **Thinking and Feeling:** Encompasses public perceptions of disease risk and vaccine confidence, including perceived benefits, safety concerns, and responses to vaccine-related information.
2) **Social Processes:** Focuses on social influences, including family support, community norms, health worker recommendations, and gender equity in vaccination access.
3) **Motivation:** Addresses individual intention and willingness to get recommended vaccine.
4) **Practical Issues:** Covers structural factors affecting vaccination, including service availability, accessibility, appointment processes, and quality of healthcare delivery.

We will design the HPV vaccine knowledge base for the chatbot-based intervention based on the BeSD framework. Through interactive dialogues with the chatbot, parents of adolescents will learn about HPV vaccine-related information, which aim to activate their vaccination drivers and enhance their vaccination willingness and behavior.

2. Development of the HPV vaccine chatbot as an intervention tool

1) Development of the HPV vaccine chatbot

In collaboration with the University of Hong Kong (HKU) team, we are developing a HPV vaccine chatbot based on ChatGPT, a generative AI released by OpenAI in late 2022. We chose ChatGPT as our foundation because of its proven capabilities in natural language understanding and health-related communications, particularly its ability to process vaccine-related queries and serve as a reliable source of public health information.

Our research methodology consists of two phases. In phase I, we will develop and validate an HPV vaccine chatbot based on ChatGPT. This involves formulating HPV vaccine questions in alignment with the BeSD theory, evaluating ChatGPT's responses for relevance and scientific accuracy, and refining these responses through expert consultation. Following the clinical trials of this ChatGPT-based chatbot, we will proceed to phase II, we will develop a localized HPV vaccine chatbot using Chinese large language models (LLMs), such as Baidu's Ernie Bot and Fudan University's MOSS.

2) Interactive service formats of the HPV vaccine chatbot

The HPV vaccine chatbot will be accessible to users via a web platform and will provide services in two formats:

- **Conversational interface**: Users can type questions about the HPV vaccine to the chatbot, and the chatbot will respond with answers presented in text, images, or tables.
- **Navigational menu**: Users can browse through hierarchical categories of HPV vaccine-related topics, clicking through preset navigation options until they identify a specific question of interest. The chatbot will then provide answers in text, images, or tables.

Whether using the conversational or navigational service format, the chatbot will offer users a link to schedule an HPV vaccination, streamlining the appointment booking process.

3. Intervention implementation phase: Evaluating the impact of the chatbot intervention on parents' HPV vaccine knowledge, vaccine confidence, willingness to vaccinate their daughters, and their daughters' HPV vaccination uptake through a cluster randomized controlled trial

1) Intervention study design

A cluster randomized controlled trial (cRCT) will be conducted, with participants randomly assigned to either the chatbot intervention group or the control group. The

questionnaire, designed based on the Behavioural and Social Drivers of Vaccination (BeSD) theory, will be administered both before and after the intervention to assess the chatbot's impact (see Figure 1). The chatbot intervention will be accessible only to the intervention group for a two-week period to minimize confounding factors and measure intervention outcomes.

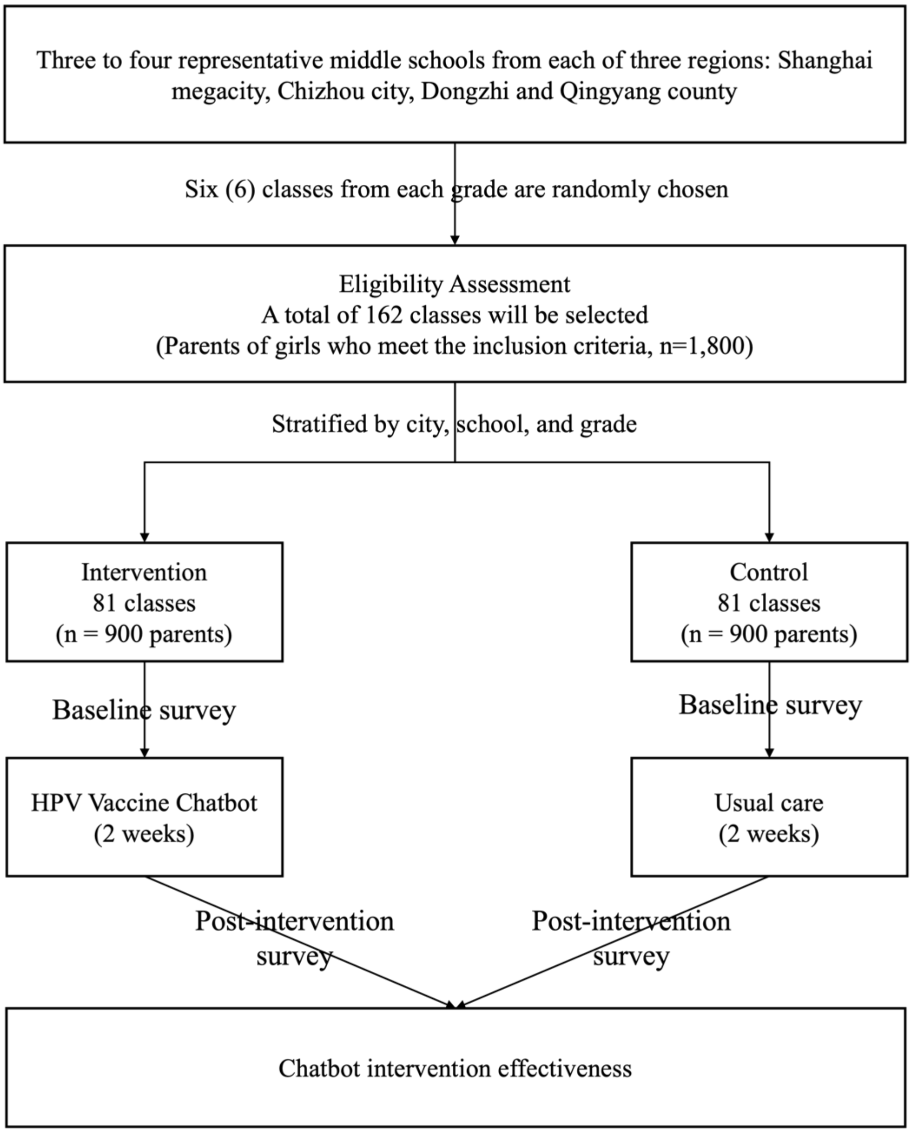

Figure 1. Flowchart of cRCT design

2) Study participants and sample size

Participants: Parents of middle school girls from three economically diverse regions in China: Shanghai (megacity), Guichi District of Chizhou City in Anhui Province (urban), and Dongzhi and Qingyang Counties of Chizhou City (rural).

Inclusion criteria: (1) Parents of middle school girls in participating schools; (2) their daughters had not received an HPV vaccine, did not have an HPV vaccination appointment scheduled and have no contraindications for HPV vaccination; (3) parents have access to a mobile device; (4) No mental disorders or visual/reading impairments, and able to participate in the intervention activities; (5) Provided informed consent and willing to participate in the study. Participants were excluded if they failed to meet any of the above inclusion criteria.

Sample size: The sample size calculation was based on the primary outcome measure (vaccination rate) and the main analysis method (comparing pre- and post-intervention rates). Based on literature review, the current HPV vaccination rate among the girls aged 9-14 years is around 5%. We assumed that the vaccination rate could increase to between 10% and 12% following the intervention. Using the formula for cluster randomized trials, the minimum sample size was calculated to be between 369 and 648 participants in each group, with a significance level (α) of 0.05, a test power (1-β) of 0.8, and a cluster design effect (DE) of 1.5. To ensure robust results and account for possible variations, each group will be set at 900 participants, with a total sample size of 1,800.

Sampling and grouping: Cluster sampling will be employed, considering factors such as economic development, school size, and geographical location. Three to four middle schools will be selected from each city, with approximately six classes randomly chosen from each grade. For each selected class, we will invite one parent of each eligible female student to participate. Parents of all eligible girls within these classes will be included, with a total of about 162 classes. Stratified cluster randomization will be conducted at a 1:1 ratio based on city, school, and grade, with 81 classes in the intervention group and 81 in the control group. Each city will have about 600 parents participating (300 in the intervention group and 300 in the control group) for a total sample size of 1,800, meeting the sample size requirements.

3) Baseline survey

Following the recruitment of participants, a baseline survey will be conducted. The questionnaire, designed based on the BeSD theory, will cover the following aspects (baseline and follow-up questionnaires are provided in the appendix):

- **Demographic variables:**
  - Parents' gender, age, education level, occupation, household income;
  - Their daughters' age and sexual health education status, etc.
- **Contextual factors:**
  - Family history of HPV vaccination;
  - Prior exposure to negative information about the HPV vaccine.
- **Primary outcome variables:**

- HPV vaccination status and appointment among participants' daughters (whether their daughters get vaccinated against HPV or have an appointment for vaccination)

- **Secondary outcome variables:**
  - Parental willingness to vaccinate their daughter (whether parents are willing to vaccinate their daughters with HPV vaccine);
  - Healthcare consultation (whether parents consulted a healthcare professional about getting the HPV vaccine for their daughters);
  - HPV vaccine awareness and knowledge (to be assessed through six HPV vaccine knowledge-related questions and four rumor-related questions);
  - Vaccine confidence (to be measured using Vaccine Confidence Index [VCI] to assess perceived importance, effectiveness, and safety).

4) Intervention

Participants will be recruited through schools. Parents in the intervention group will use the HPV vaccine chatbot via WeChat, where they can ask any questions related to the HPV vaccine. The intervention will last two weeks, and participants will receive reinforcement messages every four days. Parents in the control group will not use the chatbot. The frequency and duration of chatbot use by participants in the intervention group will be recorded.

5) Post-intervention Survey

After the two-week intervention, a follow-up survey will be conducted using the similar questionnaire to the baseline survey. For participants in the intervention group, a chatbot usability survey scale will be also used to assess chatbot usability. Participants' attitudes, evaluations, user experience, and suggestions regarding the chatbot will be collected to inform future improvements. Female middle school students' vaccination status and appointment will be obtained through vaccination clinics and the vaccination registration system.

6) Data analysis

Between-group comparison of baseline characterises

The individual characteristics of parents and their daughters will be compared between the intervention and control groups.

(1) For continuous variables, normality will be assessed. If normality is met, comparisons will be conducted using t-tests and analysis of variance (ANOVA); conversely, if not, rank-sum tests will be used.

(2) For categorical variables, comparisons will be performed using chi-square tests or Fisher's exact tests.

Outcome measures

(1) Primary outcome: HPV vaccination status and appointment among participants' daughters (whether their daughters get vaccinated against HPV or have an appointment for vaccination, measured by self-report and validated by vaccination information system)

(2) Secondary outcome measures: Parental willingness to vaccinate their daughter, parental consultation with healthcare professionals about HPV vaccination for their daughters, HPV vaccine awareness and knowledge, vaccine confidence, and chatbot usage data.

Intervention effectiveness analysis

(1) **Primary outcome analysis**:

The primary outcome, HPV vaccination status and appointment among participants' daughters (whether their daughters get vaccinated against HPV or have an appointment for vaccination system), will only be measured post-intervention. A chi-square test (or Fisher's exact test) will be used to compare differences between the intervention and control groups after the intervention, with logistic regression adjusting for potential confounding variables.

(2) **Secondary outcome analysis**:

The secondary outcome, defined as whether parents consulted a healthcare professional about getting the HPV vaccine for their daughters, will also be measured only post-intervention and analyzed using the same analytical approach as that for the primary outcome.

Other secondary outcomes will be measured before and after the intervention. Initial comparisons will be made between the intervention and control groups for each variable, focusing on pre- and post-intervention differences. For continuous variables like vaccine knowledge, t-tests will be used, while categorical variables will be analyzed with chi-square tests. Then, a difference-in-differences (DID) design will be applied to compare changes between the intervention and control groups before and after the intervention to assess the effectiveness of the chatbot intervention (Table 1).

Table 1 Difference-in-Differences Method

Grouping	Pre-intervention	Post-intervention	Difference (D)	Difference-in-Differences (DID)
Intervention Group (T)	$y_{0,T}$	$y_{1,T}$	$y_{1,T} - y_{0,T}$	$(y_{1,T} - y_{0,T}) - (y_{1,C} - y_{0,C})$
Control Group (C)	$y_{0,C}$	$y_{1,C}$	$y_{1,C} - y_{0,C}$	

The difference-in-differences (DID) model is as follows:

$$Y_{it} = \alpha + \beta_1 Time_t + \beta_2 Treat_i + \delta(Time_t \times Treat_i) + \epsilon_{it}$$

where Y_{it} represents the outcome variable for individual i at time t, and α is the constant term. $Time_t$ and $Treat_i$ are the time and intervention variables, respectively. When $t = 1$, it indicates the post-intervention period, and when $t = 0$, it indicates the pre-intervention period. When $i = 1$, it denotes the intervention group, and when $i = 0$, it denotes the control group. The term $Time_t \times Treat_i$ represents the interaction between the intervention and time variables. β_1 and β_2 are the coefficients for the time and intervention variables, respectively, while δ is the coefficient for the interaction term, which is the key parameter of interest in this study, representing the intervention effect. ϵ_{it} represents unobserved factors that may influence the outcome variable.

Thus, the interaction term between the time and intervention variables is included in the model to assess the intervention effect. A mixed-effect model will be used for continuous variables, such as HPV-related knowledge. For categorical variables, such as HPV vaccination status and appointment, parents' consultation with healthcare providers, HPV vaccine confidence, and their willingness to vaccinate their daughters, a generalized estimating equation (GEE) will be employed.

4. Promotion phase: assessing chatbot acceptability and scalability through qualitative interviews, optimizing the chatbot, and expanding its application

After the intervention phase, we will conduct follow-up surveys to assess the chatbot's acceptability and scalability.

(1) **Assessing chatbot acceptability through focus group discussions**

We will randomly select one parent of middle school girls from each grade at each school for group interviews. The group discussions will focus on evaluating the chatbot's acceptability, user experience, feedback, as well as participants' preferences for the chatbot's service delivery and content, and their expectations for additional features or services.

(2) **Assessing chatbot scalability through interviews with public health professionals**

We will conduct interviews with eight professionals in each location, including staff from local Centers for Disease Control and Prevention and vaccination sites. The interviews will cover their evaluation of the chatbot, suggestions for improvements based on trial outcomes, and recommendations for its broader implementation.

(3) **Optimizing the chatbot and expanding its adoption**

We will refine the chatbot's service content and interface design based on the findings from the user and professional interviews. The chatbot will then be expanded to include participants from the control group, as well as individuals from other schools and vaccination sites within the local area. Subsequently, we will select ten parents from the control group and eight health professionals from vaccination sites for follow-up interviews. The interviews will remain the same as previously described.

5. Evaluating the applicability and scalability of the chatbot intervention through implementation science

We will conduct an implementation science study, guided by RE-AIM framework (Reach, Effectiveness, Adoption, Implementation, and Maintenance), to assess the cost-effectiveness, applicability, and scalability of the HPV vaccine chatbot intervention in real-world settings. This study will employ a mixed-methods approach, integrating both quantitative and qualitative methods, to evaluate the intervention across five dimensions: reach, effectiveness, adoption, implementation quality, and sustainability (Table 2).

Table 2 Implementation Science Evaluation of the HPV Vaccine Chatbot Intervention Based on the RE-AIM Framework

Dimensions	Evaluation Indicators	Evaluation Timing and Methods
**Reach:** Proportion of the target population engaged	1. Number and proportion of parents willing to participate in the HPV vaccine chatbot intervention;<br>2. Total number of interactions with the chatbot.	During the intervention implementation phase (chatbot backend data and surveys)
**Effectiveness:** Impact of the intervention on outcomes	Change in parents' knowledge, confidence, willingness to vaccinate, and behaviors regarding the HPV vaccine, as measured before and after the intervention (see Section 3.3).	During the intervention implementation phase (surveys and vaccination information system records)
**Adoption:** Proportion of organizations and individuals adopting the intervention	1. Results from a chatbot usability survey assessing its usability and acceptability.<br>2. Number and proportion of parents and healthcare institutions adopting the chatbot as a health education tool.<br>3. Number and proportion of parents willing to recommend the chatbot to others.<br>4. Reasons for adoption or non-adoption by parents and organizations, and their future willingness to adopt and use the chatbot.	During the intervention implementation and promotion phases (surveys, parent and professional interviews)
**Implementation:** Fidelity and quality of the intervention implementation	**Fidelity:**<br>1. Proportion of participants actively engaging in chatbot conversations.<br>2. Average duration of interactions and total number of interaction questions raised during chatbot consultations.<br>**Implementation Issues:**<br>1. Assessment of chatbot's content, design, and overall acceptability.	During the intervention implementation phase (chatbot backend data, parent and professional interviews)
	**At the organizational level:**	

**Maintenance:** Long-term sustainability of the intervention	1. Routine adoption of the chatbot as a health education tool by organizations and vaccination personnel.<br>**At the public level:**<br>1. Long-term follow-up on whether parents continue to use the chatbot, including frequency of use and trends in user proportion over time.<br>2. Long-term adolescent HPV vaccination coverage.	During the promotion phases (chatbot backend data, vaccination information system records)

1) Implementation science evaluation data sources

The implementation science evaluation will cover both the intervention implementation stage and the promotion stage, adopting both quantitative and qualitative methods. Quantitative data will be collected from chatbot interaction logs, intervention-phase parent questionnaires, and local immunization registry systems. Qualitative data will be gathered during the promotion stage through in-depth interviews and focus group discussions with three stakeholder groups: CDC staff, community health service center staff (both directors and healthcare providers) and parents.

2) Implementation science evaluation methods

- **Quantitative Evaluation:** Descriptive statistical analysis, including means (standard deviations) and frequencies (percentages), will be used to describe the chatbot intervention's coverage, adoption, implementation, and sustainability.
- **Qualitative Analysis:** Using structured qualitative methods and thematic framework analysis, we will examine factors influencing chatbot coverage and adoption, identify implementation barriers and challenges, and assess value for wider implementation and recommendations.

6. Timeline

Project Duration: September 2023 to December 2025

September 2023 to December 2024: Protocol development, field work initiation, baseline assessment, intervention implementation, follow-up assessment

January to December 2025: Manuscript preparation, report writing, and result dissemination

7. Study setting

The study will be conducted at:

- Shanghai Megacity: Zhenchuan Middle School, Jinhe Middle School, Sumin Middle School, and Liucheng Experimental Middle School;

- Chizhou City, Anhui Province: No. 10 Middle School, No. 11 Middle School, and No. 16 Middle School;
- Dongzhi and Qingyang County, Anhui Province: Lanxi Middle School at Dongzhi County, Yaocheng Middle School at Dongzhi County, and Qingyang No. 4 Middle School.

8. Research team

(1) Team leaders:

Leesa Lin, PhD, Assistant Professor

1 Department of Infectious Disease Epidemiology and Dynamics, London School of Hygiene & Tropical Medicine, London, United Kingdom

2 Laboratory of Data Discovery for Health Limited ($D^2$4H), Hong Kong Science Park, Hong Kong SAR, Hong Kong 999077, China

3 WHO Collaborating Centre for Infectious Disease Epidemiology and Control, School of Public Health, Li Ka Shing Faculty of Medicine, The University of Hong Kong, Sassoon Road, Hong Kong SAR, Hong Kong 999077, China

Email: Leesa.Lin@lshtm.ac.uk

Zhiyuan Hou, PhD, Associate Professor
4 School of Public Health, Fudan University, Shanghai 200032, China
5 National Health Commission Key Laboratory of Health Technology Assessment, Fudan University, Shanghai 200032, China

Email: zyhou@fudan.edu.cn

(2) **Team members:**

- Fudan University: **Zhiyuan Hou**, Associate Professor; **Zhiqiang Qu**, Master Student
- Laboratory of Data Discovery for Health Limited, Hong Kong Science Park, and School of Public Health, The University of Hong Kong: **Leesa Lin**, Assistant Professor; **Joseph T. Wu**, Professor; **Zhengdong Wu**, Master Student
- London School of Hygiene & Tropical Medicine: **Leesa Lin**, Assistant Professor; **Heidi J. Larson**, Professor; **Mark Jit**, Professor
- Local CDCs: **Liubing Gong**, Associate Director of Chizhou Center for Disease Control and Prevention, Anhui Province; **Hui Peng**, Director of School Health Department, Jiading District Center for Disease Control and Prevention, Shanghai

Reference

[1] Behavioral and social drivers of vaccine uptake: tools and guidance for achieving high uptake. Geneva: World Health Organization; 2022.

[2] World Health Organization. Human papillomavirus vaccines: WHO position paper (2022 update). Weekly Epidemiological Record. 2022, 97 (50), 645-672.

[3] Hu S, Xu X, Zhang Y, et al. A nationwide post-marketing survey of knowledge, attitude and practice toward human papillomavirus vaccine in general population: Implications for vaccine roll-out. Vaccine. 2021 Jan 3;39(1):35-44.

[4] 宋祎凡,刘晓雪,尹遵栋,等.2018-2020 年中国 9-45 岁女性人乳头瘤病毒疫苗估算接种率 [J].中国疫苗和免疫,2021,27(05):570-575.

[5] 邱丽蓉,牛战琴.9～14 岁女性人乳头瘤病毒疫苗接种现状及其影响因素分析[J].中国生育健康杂志,2022,33(03):262-265.

[6] Falcaro M, Castanon A, Ndlela B, et al. The effects of the national HPV vaccination programme in England, UK, on cervical cancer and grade 3 cervical intraepithelial neoplasia incidence: a register-based observational study. Lancet 2021; 398(10316): 2084-92.

[7] Drolet M, Benard E, Perez N, Brisson M, Group HPVVIS. Population-level impact and herd effects following the introduction of human papillomavirus vaccination programmes: updated systematic review and meta-analysis. Lancet 2019; 394(10197): 497-509.

[8] Sonawane K, Zhu Y, Montealegre JR, et al. Parental intent to initiate and complete the human papillomavirus vaccine series in the USA: a nationwide, cross-sectional survey. Lancet Public Health 2020; 5(9): e484-e92.

[9] Zang S, Zhang X, Qu Z, et al. Promote COVID-19 Vaccination for Older Adults in China. China CDC Wkly 2022; 4(37): 832-4.

[10] 史金晶,张肖肖,郑徽,等.中国大陆青少年家长人乳头瘤病毒疫苗认知度和接受度 Meta 分析[J].中国疫苗和免疫,2019,25(04):464-470.

[11] 武丽,马远珠,黄雪萍,等.广东省青少年女性对 HPV 疫苗知晓及接种意愿的调查研究[J]. 中国妇幼卫生杂志,2021,12(05):19-23.

[12] Zhang Y, Wang Y, Liu L, et al. Awareness and knowledge about human papillomavirus vaccination and its acceptance in China: a meta-analysis of 58 observational studies. BMC Public Health 2016; 16: 216.

[13] Xie Y, Su LY, Wang F, Tang HY, Yang QG, Liu YJ. Awareness regarding and vaccines acceptability of human papillomavirus among parents of middle school students in Zunyi, Southwest China. Hum Vaccin Immunother 2021; 17(11): 4406-11.

[14] Wei Z, Liu Y, Zhang L, et al. Stages of HPV Vaccine Hesitancy Among Guardians of Female Secondary School Students in China. J Adolesc Health. 2022. Epub 2022/10/14.

[15] Zhang X, Wang Z, Ren Z, et al. HPV vaccine acceptability and willingness-related factors among Chinese adolescents: a nationwide study. Hum Vaccin Immunother 2021; 17(4): 1025-32.

[16] Zhang X, Liu CR, Wang ZZ, et al. Effect of a school-based educational intervention on HPV and HPV vaccine knowledge and willingness to be vaccinated among Chinese adolescents: a multi-center intervention follow-up study. Vaccine 2020; 38(20): 3665-70.13

[17] Wang D, Wu J, Du J, et al. Acceptability of and barriers to human papillomavirus vaccination in China: A systematic review of the Chinese and English scientific literature. Eur J Cancer Care (Engl) 2022; 31(3): e13566.

[18] Si M, Su X, Jiang Y, et al. Effect of an IMB Model-Based Education on the Acceptability of HPV Vaccination Among College Girls in Mainland China: A Cluster RCT. Cancer Control 2022; 29: 10732748211070719.

[19] Si M, Su X, Jiang Y, et al. An Internet-Based Education Program for Human Papillomavirus Vaccination Among Female College Students in Mainland China: Application of the Information-Motivation-Behavioral Skills Model in a Cluster Randomized Trial. J Med Internet Res. 2022 Sep 30;24(9):e37848.

[20] Luk TT, Lui JHT, Wang MP. Efficacy, Usability, and Acceptability of a Chatbot for Promoting COVID-19 Vaccination in Unvaccinated or Booster-Hesitant Young Adults: Pre-Post Pilot Study. J Med Internet Res 2022; 24(10): e39063.

[21] Kathy Leung, Kristi Lee, Saudamini Dabak et al. The effectiveness of conversational AI services on Covid-19 vaccine confidence and acceptance in Thailand, Hong Kong, and Singapore, 22 December 2022, PREPRINT (Version 1) available at Research Square [https://doi.org/10.21203/rs.3.rs-2127117/v1]

[22] Wilson L, Marasoiu M. The Development and Use of Chatbots in Public Health: Scoping Review. JMIR Hum Factors. 2022;9(4):e35882.

[23] Almalki M, Azeez F. Health Chatbots for Fighting COVID-19: a Scoping Review. Acta Inform Med 2020; 28(4): 241-7.

[24] Kobayashi T, Nishina Y, Tomoi H, et al. Corowa-kun: A messenger app chatbot delivers COVID-19 vaccine information, Japan 2021. Vaccine 2022; 40(32): 4654-62.

[25] Weeks R, Cooper L, Sangha P, et al. Chatbot-Delivered COVID-19 Vaccine Communication Message Preferences of Young Adults and Public Health Workers in Urban American Communities: Qualitative Study. J Med Internet Res 2022; 24(7): e38418.

Supplementary Document 2: Study protocol for HPV vaccine chatbot (Chinese version)

开发疫苗聊天机器人干预并评估其对我国 HPV 疫苗信心的影响

研究方案

（终版）

侯志远课题组

复旦大学公共卫生学院

2024 年 2 月 18 日

复旦大学公共卫生学院
School of Public Health, Fudan University
138 Yi Xue Yuan Road
Shanghai 200032, China

开发疫苗聊天机器人干预并评估其对我国 HPV 疫苗信心的影响

摘要：接种人乳头瘤病毒(HPV)疫苗是预防宫颈癌最具经济效益的措施。作为全球第二大宫颈癌负担国，我国 HPV 疫苗接种率非常低，最应优先接种的青少年接种率更低。主要障碍之一是青少年家长对 HPV 疫苗认知不足；然而传统干预作用有限，亟需探索有效干预来改善青少年家长 HPV 疫苗认知和接种。本研究基于人工智能技术开发 HPV 疫苗聊天机器人，在青少年家长中开展聊天机器人干预，通过整群随机对照试验评估其对 HPV 疫苗知识、认知、接种意愿和行为的影响；通过实施科学研究评价聊天机器人干预项目实施的可接受性、可推广性、可持续性，提升聊天机器人在公共卫生领域的应用价值。建立的疫苗聊天机器人平台，可用于高效的 HPV 疫苗信息沟通交流，成为 HPV 疫苗的接种干预工具。

一、研究背景与趋势

（一）研究的重要意义

宫颈癌在全球造成极大的健康危害，接种人乳头瘤病毒(Human Papillomavirus, HPV)疫苗是预防宫颈癌最具经济效益的措施。世界卫生组织推荐将 HPV 疫苗纳入免疫规划，建议优先为 9-14 岁女孩接种。然而，作为全球第二大宫颈癌负担国，我国 HPV 疫苗接种率非常低，最应优先接种的青少年接种率更低。青少年家长对 HPV 疫苗认知不足是主要障碍之一，传统干预措施作用有限，亟需探索有效干预来改善其 HPV 疫苗相关认知、信心和接种行为。与传统干预措施相比，新冠疫情期间兴起的智能聊天机器人提供了改善公众 HPV 疫苗认知的新途径，需要评估其效果和应用价值。

1、我国 HPV 疫苗接种率低，尤其最应该保护的青少年接种严重不足

接种 HPV 疫苗可以有效预防宫颈癌，并且现有证据显示接种年龄越早预防效果越好。世界卫生组织建议将 HPV 疫苗纳入免疫规划，优先为 9-14 岁女孩接种，并在 2020 年提出了《加速消除宫颈癌全球战略》。中国也加入了此战略，在 2023 年 1 月印发了《加速消除宫颈癌行动计划（2023-2030 年）》，提出要在未来 10 年内试点推广、持续推进适龄女孩 HPV 疫苗接种服务，并探索多渠道支持适龄女孩 HPV 疫苗接种。然而，我国目前尚未将 HPV 疫苗纳入国家免疫规划，并且前期我国 HPV 疫苗接种主要关注成年人和大学生群体，对接种最具成本效益的青少年反而重视不足。据估算，2018-2020 年中国 9-45 岁适龄女性 HPV 疫苗累计接种率仅为 2.24%，而青少年接种率更是不足 2%。

2、青少年及家长对宫颈癌和 HPV 疫苗认知不足，严重阻碍了青少年的 HPV 疫苗接种

阻碍青少年 HPV 疫苗接种的原因包括疫苗供给不足、价格昂贵、公众认知不足等。目前，我国多款 HPV 疫苗产品获批，越来越多的地区开始青少年免费接种，解决了供给和价格问题。然而，我国青少年性健康教育严重滞后，对人乳头瘤病毒感染等性传播疾病及其导致的宫颈癌缺乏认知，缺乏专业渠道获取 HPV 疫苗相关知识；而网上信息鱼龙混杂，关于 HPV 疫苗的谣言也很多，容易影响青少年及其家长的正确认知。不知晓 HPV 疫苗、感知不到宫颈癌疾病风险、感知不到接种疫苗的必要性/有效性在青少年及其家长群体中尤为突出，

成为青少年HPV疫苗接种的主要障碍。另外，九价HPV疫苗在2022年9月扩龄之前在我国仅被批准用于16-26岁的女性；部分人群对九价疫苗的执念也一定程度延迟了青少年的疫苗接种。

3、与传统干预措施相比，新冠疫情期间兴起的智能聊天机器人提供了改善公众HPV疫苗认知的新途径，需要评估其效果和应用价值。

改善疫苗认知的传统干预措施包括健康教育、专业讲座、医务人员推荐等，这些传统干预措施均是公众被动接受干预内容的单向输出，缺乏互动，不能充分满足公众需求。由于国内长期存在的医患矛盾大环境，医务人员也普遍不太愿意向公众推荐HPV疫苗等二类疫苗。而且，传统干预措施因成本高、实施周期长，可推广性和可持续性有限。

新冠疫情期间，基于AI技术的聊天机器人在公共卫生领域逐渐兴起。相比于传统被动干预，聊天机器人互动式的干预形式更能满足公众对健康信息的主观需求；并且聊天机器人往往基于社交媒体、网络等载体，可实施性和推广性强。作为方便可及的交互式健康信息资源，聊天机器人可以应用到HPV等疫苗领域。聊天机器人以公众需求为导向而向其提供疫苗信息、支持其疫苗接种决策，是潜在的疫苗接种助推工具。因此，需要开发HPV疫苗聊天机器人，评估其对HPV疫苗认知的影响，探讨其在疫苗领域的应用价值。

本研究拟解决的问题如下：

1) 如何开发一款HPV疫苗聊天机器人，保证对话信息的适用性和科学性？并评估其作为HPV疫苗信心干预工具的可行性？
2) 聊天机器人干预对促进青少年家长HPV疫苗相关知识、认知、信心和行为的效果如何？影响干预效果的因素有哪些？
3) 各方对疫苗聊天机器人的接受度如何？聊天机器人干预项目实施科学性和可推广性如何？

（二）研究的创新点

本研究有以下三个特色和创新点：

1. 利用人工智能和公共卫生交叉研究，基于人工智能技术开发用于促进HPV疫苗接种的聊天机器人，提高疫苗信息沟通效能；
2. 评估HPV疫苗聊天机器人的对话表现和作为干预工具的可行性，并开展HPV疫苗聊天机器人干预，评估聊天机器人对HPV疫苗接种的影响和应用价值；
3. 利用实施科学方法，全面评估HPV疫苗聊天机器人干预项目在真实世界中的可接受性、适用性和可推广性。

（三）相关领域研究现状与趋势（含文献综述）

1、我国青少年及其家长对宫颈癌和HPV疫苗的认知现状

我国于2016年首次引进HPV疫苗，截至2022年，中国共有5种HPV疫苗上市，分别是葛兰素史克的二价疫苗、默沙东的四价和九价疫苗，以及厦门万泰和沃森生物的两款国产二价疫苗。HPV疫苗在国内已上市6年，然而接种状况并不乐观，适龄女性接种率仅为2.24%，其中，对接种HPV疫苗获益最大的9-14岁女孩接种率不到2%。

1）青少年对宫颈癌和HPV疫苗的认知现状

除供应不足、价格过高外，HPV疫苗相关认知不足是影响青少年HPV疫苗接种的重要因素，如宫颈癌疾病风险感知不足、HPV疫苗相关知识不足、对疫苗安全性存在顾虑等。2016年的调查中，全国青少年宫颈癌知晓率仅32.8%，70%不觉得自己有发生宫颈癌的风险，仅12.9%和17.1%听说过HPV和HPV疫苗。到2020年，青少年HPV疫苗知晓度有所改善，达65%，但知晓改善的主要是大学生（79%），10-14岁学生的知晓率与接种意愿仍然最低，分别为23%和43%。不了解HPV疫苗是不愿接种的最主要的原因（76%）。

2）青少年家长对宫颈癌和HPV疫苗的认知现状

家长对HPV疫苗的认识和接受度会影响青少年HPV疫苗的接种，而国内家长对HPV疫苗相关认知不容乐观。一项涵盖国内19个省份的荟萃分析显示，中国居民HPV疫苗平均知晓率仅16%，为子女接种意愿约60%。另外一项荟萃分析中，中国大陆青少年家长对HPV及其疫苗的知晓率分别仅为28%、19%，为子女接种意愿为41%。另一方面，存在不少谣言影响公众HPV疫苗认知，很多家长盲目追求高价次HPV疫苗而导致青少年接种延迟，错过最佳接种年龄。

2、影响HPV疫苗认知和接种的干预措施现状和趋势

1）传统干预措施现状

疫苗领域的传统干预方式包括健康教育、推广讲座、医务人员推荐等，一定程度能改善青少年HPV疫苗相关认知和接种意愿。但是传统干预存在一些限制，例如，均以受试者被动接受干预内容为主，无法满足主观需求；干预成本高、周期长；大多属于一过性干预，推广难度大、可持续性受限等。要快速地全面推广HPV疫苗接种需要探索更为高效可行的干预措施。

2）新兴干预措施趋势——聊天机器人

新冠疫情大流行中，疫苗聊天机器人的应用研究逐渐兴起。新冠疫苗聊天机器人主要通过提供以下信息而支持用户接种决策：（1）疾病相关知识，如易感性、严重性；（2）疫苗相关知识，如重要性、安全性、有效性等；（3）疫苗接种资质判断；（4）人群接种现状；（5）如何预约接种；（6）接种建议，如接种前/后的注意事项；（7）抗击相关谣言与负面新闻。香港地区2021年底的研究发现聊天机器人可以有效降低新冠疫苗犹豫、增加疫苗信心并促进接种，在拒绝或犹豫接种的人群中，聊天机器人干预后新冠疫苗犹豫程度降低了14.7%，新冠疫苗及加强针的接种意愿分别增加了30%和47.4%。香港地区和美国的研究均报告了公众对聊天机器人的高接受度（>70%），尤其在抗击谣言和负面新闻方面效果显著。目前国外已发布的疫苗聊天机器人包括美国霍普金斯大学发布的VIRA、日本学者发布的Corowa-kun、泰国公共卫生部发布的ChatSure等。尤其2022年11月OpenAI人工智能公司发布了旗下聊天机器人产品ChatGPT，其强大的对话能力引起了业界和学术界的广泛讨论和应用研究。聊天机器人载体可变，具有非常强的可实施性和可推广性。因此在疫苗领域，相较于传统干预，聊天机器人干预具有不可替代的成本低、可持续性强等优势，潜在应用价值巨大。

3、国内HPV疫苗接种相关干预研究缺乏，聊天机器人相关研究尚为空白

目前国内HPV疫苗研究集中在接种率与接种意愿的评估，鲜有进行干预的实证研究，截至目前也尚未发现聊天机器人应用于疫苗领域的相关研究。而聊天机器人在国际上颇受欢迎，新冠疫情大流行中，国外学者通过将聊天机器人集成在WhatsApp、Facebook、Line等社交媒体平台，已开展一系列研究助力疫情防控，包括利用聊天机器人促进新冠疫苗接种、

进行个人风险评估与评级、健康症状监测等。社交媒体和网络的普及为聊天机器人的便利性和可及性提供了条件，国内也拥有微信、抖音等用户广泛的社交媒体，具有联合开发聊天机器人的潜力。但是目前国内应用聊天机器人促进疫苗接种的研究较为缺乏，聊天机器人在国内是否有效、如何全面评估其在国内疫苗领域的应用价值亟需进一步研究。

二、研究方向和重点内容

1、研究方向

基于人工智能（AI）技术，开发用于促进 HPV 疫苗接种的聊天机器人，验证其用于 HPV 疫苗信息干预的适用性、科学性，并通过整群随机对照试验评估其对 HPV 疫苗认知、接种意愿和行为的干预效果，探索聊天机器人的可接受性和推广性，提升聊天机器人在公共卫生领域的应用价值。

2、重点研究内容

1）设计和开发 HPV 疫苗聊天机器人，作为 HPV 疫苗接种的干预工具

我们将在疫苗接种的行为社会驱动因素理论指导下，设计用于聊天机器人的 HPV 疫苗问题答案库。先基于 ChatGPT 开展 HPV 疫苗聊天机器人开发试验，ChatGPT 是一款对话式的成熟机器人，我们将验证 ChatGPT 回答 HPV 疫苗相关问题的适用性与科学性，改进其回答欠佳或无法回答的 HPV 疫苗相关问题答案，形成 HPV 疫苗聊天机器人。开发试验成功后，后续将基于国内生成式预训练模型开发独立的、成熟的 HPV 疫苗聊天机器人。

2）开展聊天机器人干预，评估其对青少年家长 HPV 疫苗认知、接种意愿和行为的影响

利用所开发的 HPV 疫苗聊天机器人，在上海市、安徽省池州市贵池区（一般城市）、池州市东至县和青阳县（农村）三地开展干预实验。干预对象为初中女生的父母，采用整群随机对照试验，在干预前后开展两轮调查，评估聊天机器人干预对 HPV 疫苗认知、接种意愿和行为的影响，为全面评估聊天机器人对促进 HPV 疫苗接种的应用价值提供依据。

3）评估聊天机器人的可接受性和推广性，探索扩展其在公共卫生领域的应用范围

开展定性访谈，探讨用户对聊天机器人的接受度、使用感受、服务偏好和意见，了解专业工作人员对聊天机器人的改进评价和推广建议，根据访谈结果，优化聊天机器人；通过实施科学方法，系统评估整个干预项目的可推广性。综合上述效果评估证据和实施科学证据，探讨其在流感疫苗等其他疫苗领域以及更广泛的公共卫生领域使用的可行性。

三、研究方法与技术路线

1、聊天机器人 HPV 疫苗信息设计与干预理论

聊天机器人上的 HPV 疫苗信息的设计，将基于疫苗接种的行为社会驱动因素理论（Behavioural and Social Drivers of Vaccination, BeSD 理论）。该模型由世界卫生组织于 2022 年提出，将影响疫苗接种的行为和社会驱动因素概括为 4 个模块：

1）公众的看法和感受，即对疫苗可预防疾病及其疫苗的认知和情绪反应，如对疾病的

风险感知、对疫苗益处/安全性的信心、相关负面新闻等；

2）影响个人决策的社会历程，即关于疫苗接种和接受疫苗接种建议的社会规范，如他人支持/建议、疫苗接种规范、社会规范、工作场所规范等；

3）接种动机，即接种疫苗的意愿和犹豫程度；

4）接种的实际问题，即接种疫苗时的经历与障碍，如知晓接种地点和预约方式等疫苗接种相关服务、疫苗可及性、接种地点适宜性等。

以 BeSD 理论为指导，我们将根据疫苗接种的行为和社会驱动因素来设计聊天机器人上的 HPV 疫苗信息。青少年家长通过聊天机器人进行对话互动，了解 HPV 疫苗相关信息，从而激发他们接种疫苗的驱动因素，提高接种意愿和行为。

2、在 BeSD 理论指导下开发 HPV 疫苗聊天机器人，作为 HPV 疫苗接种的干预工具

1）在 BeSD 理论指导下，开发 HPV 疫苗聊天机器人

我们正在和香港大学团队合作，基于最新的聊天机器人 ChatGPT 优化其在疫苗领域的对话交流。ChatGPT 是由人工智能研究实验室 OpenAI 在 2022 年底发布的人工智能技术驱动的全新聊天机器人，能够通过学习和理解人类的语言来进行对话交流。ChatGPT 也能够回答疫苗等健康领域的问题，可以成为公众健康信息的重要来源。

本研究中，我们将在 BeSD 理论指导下设计 HPV 疫苗相关问题，首先通过与 ChatGPT 对话来验证其所提供的 HPV 疫苗领域问题答案的适用性与科学性；然后改善 ChatGPT 回答欠佳或无法回答的 HPV 疫苗相关问题答案，以提供更完整、适宜的疫苗信息，通过专家咨询验证答案的匹配度、准确性和饱和度。我们将基于此基础，在国内 AI 技术公司的支持下形成基于国内平台的成熟的 HPV 疫苗聊天机器人。国内目前已发布了百度旗下"文心一言"、复旦大学自然语言处理实验室"MOSS"等大型语言模型（Large language model, LLM），基于 ChatGPT 的 HPV 疫苗聊天机器人开发试验成功后，我们将基于上述国内大型语言模型，开发独立的、成熟的 HPV 疫苗聊天机器人。

2）HPV 疫苗聊天机器人的互动服务形式

HPV 疫苗聊天机器人将通过网页以供用户访问，以两种形式提供服务。

- 对话式服务：用户通过文字向机器人提出 HPV 疫苗相关问题，机器人将通过文字、图片或表格提供答案。
- 导航式服务：用户通过预设的导航栏，逐层点击 HPV 疫苗相关问题分类，直至关心的最底层目标问题，机器人将通过文字、图片或表格提供答案。

无论是对话式还是导航式服务，聊天机器人都会向用户提供 HPV 疫苗接种的预约链接，以方便用户预约接种。

3、干预实施阶段：通过整群随机对照试验，评估聊天机器人干预对青少年 HPV 疫苗认知、接种意愿和行为的影响

1）干预研究设计

采用整群随机对照设计，将招募对象随机分成聊天机器人实验组和对照组，基于疫苗接种的行为社会驱动因素理论（BeSD 理论）设计调查问卷，在干预前后分别开展调查，以评价聊天机器人的干预效果（见图 2）。为了减少干预噪音，聊天机器人将内部运行两周，仅对实验组开放访问权限，用于干预效果的评价研究。

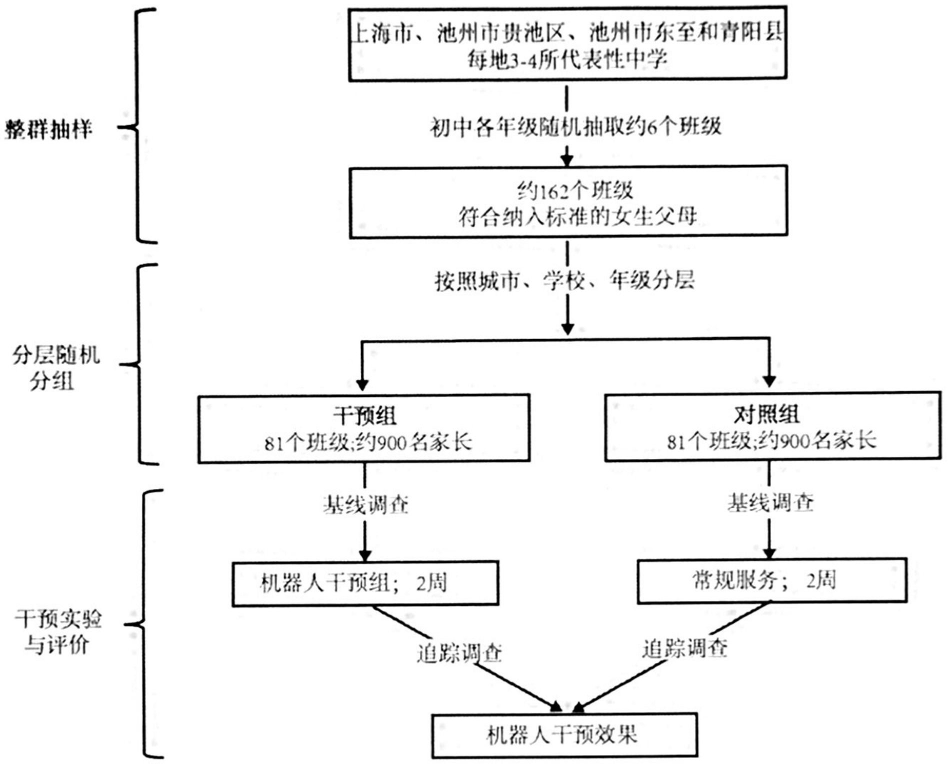

图 2 HPV 疫苗聊天机器人干预研究设计：整群随机对照试验

2）研究对象与样本

研究对象：上海市（大城市）、安徽省池州市贵池区（一般城市）、池州市东至县和青阳县（农村）的初中女学生父母。纳入标准：（1）初中女孩父母；（2）女孩无 HPV 疫苗接种禁忌症；（3）父母拥有扫码电子设备；（4）无精神障碍性疾病和视觉、阅读障碍，能够配合和实施相应干预活动；（5）取得知情同意且愿意参与研究。

样本量：根据主要结果指标（接种率）和主要分析方法（干预前后率的差异比较）计算样本量。根据文献综述，无政策干预下我国 9-14 岁女生 HPV 疫苗接种率约为 5%，我们假设干预后可提升至 10-12%，在检验水准 $\alpha=0.05$，检验效能（$1-\beta$）=0.8，整群设计效应 D=1.5 的条件下，计算得到每组最小样本量为 369-648，考虑结果稳健和可能的差异等因素，实际调查每组设定为 900 人，总样本量 1800 人。

抽样与分组：首先采用整群抽样，根据经济发展、学校规模、地理位置等因素，从每个城市选择 3-4 所中学，每所中学初中三个年级各随机选择 6 个左右班级，班级内所有符合纳

入标准女生母亲或父亲纳入研究，预计共纳入 162 个左右班级。其次采用分层整群随机分组，按照城市、学校和年级分层，随机纳入实验组和对照组各 81 个左右班级。预计每个城市约 600 名女生家长（300 干预组；300 对照组）参与研究，总样本量约 1800 人，满足样本量要求。

3）基线调查

研究对象招募后将进行基线调查，依据疫苗接种的行为社会驱动因素理论（BeSD 理论）设计调查问卷（基线及追踪调查问卷见附件），包含以下方面：

人口学变量：

家长性别、年龄、教育水平、工作、家庭年收入；女孩年龄、女孩性教育情况等；

环境变量：

家人 HPV 疫苗接种史、HPV 疫苗负面信息暴露情况等；

主要结果变量：

家长为女儿接种 HPV 疫苗的行为（是否接种/预约 HPV 疫苗）；

次要结果变量：

接种意愿（是否愿意为女儿接种 HPV 疫苗）；

HPV 疫苗医生咨询：是否咨询医务人员为女儿接种 HPV 疫苗；

HPV 疫苗知识：将设置 6 个知识性问题和 4 个谣言性问题；

疫苗信心：采用疫苗信心指数（Vaccine Confidence Index, VCI）中的疫苗重要性、安全性、有效性，调查家长感知的 HPV 疫苗重要性、安全性、有效性；

4）实施干预

通过学校邀请研究对象。实验组父母通过微信入口使用 HPV 疫苗聊天机器人，可以询问任何有关 HPV 疫苗的问题；干预两周，每 4 天邀请一次，强化干预。对照组的女孩父母均不使用聊天机器人。

干预周期共两周，记录实验组研究对象使用聊天机器人的频率和时长。

5）干预后追踪调查

两周干预结束后进行追踪调查，调查内容与基线调查一致。对实验组调查对象，还将利用**聊天机器人可用性调查量表**评估聊天机器人可用性；同时调查干预组对象对该聊天机器人的态度、评价、使用感受和建议，为后续优化聊天机器人提供依据。

通过对接接种单位和接种信息系统获得研究对象 HPV 疫苗预约和接种情况。

6）数据分析

基线资料组间可比性分析

对家长及其女儿的个人特征，将在随机分组后比较干预组和对照组的差异，以检验组间的可比性。

（1）对于连续变量，满足正态性时，将通过 T 检验、方差分析（ANOVA）进行比较分析；不满足时将通过秩和检验进行。

（2）对于分类变量，将通过卡方检验或 Fisher 精确概率法进行比较。

结果指标

（1）主要结果指标：家长为女儿接种 HPV 疫苗的行为（是否接种/预约 HPV 疫苗）

（2）次要结果指标：为女儿接种的意愿、是否咨询医务人员为女儿接种 HPV 疫苗、HPV 疫苗知识、疫苗信心、机器人使用情况。

干预效果分析

（1）主要结果指标：

主要结果指标的接种行为仅在干预后测量，将采用卡方检验（Fisher 精确概率法）比较干预组和对照组干预后的差异，并通过 Logistic 调整其他协变量的可能影响。

次要结果指标中的"是否咨询医务人员为女儿接种 HPV 疫苗"亦仅在干预后测量，采用和主要结果指标一样的分析方法。

（2）次要结果指标

次要结果指标在干预前后均测量。首先将比较每个变量干预前后、干预组和对照组之间的差异，疫苗知识为连续变量将采用 T 检验；其他变量为分类变量将采用卡方检验。其次，将采用双重差分设计，比较干预组和对照组干预前后变化的差异，评估聊天机器人干预的效果（表 1）。

表 1　聊天机器人干预效果的评价方法（倍差法）

分组	干预前	干预后	Difference (D)	Difference-in-Differences (DID)
实验组 T	$y_{0,T}$	$y_{1,T}$	$y_{1,T} - y_{0,T}$	$(y_{1,T} - y_{0,T}) - (y_{1,C} - y_{0,C})$
对照组 C	$y_{0,C}$	$y_{1,C}$	$y_{1,C} - y_{0,C}$	

双重差分设计的模型如下：

$$Y_{it}=\alpha+\beta_1 Time_t+\beta_2 Treat_i+\delta(Time_t\times Treat_i)+\epsilon_{it}$$

其中，Y_{it} 表示表示个体 i 在时间 t 的结果变量，α 为常数项，$Time_t$ 和 $Treat_i$ 分别为时间变量和干预变量 t=1 时表示干预后，t=0 时表示干预前，i=1 时表示干预组，i=0 时表示对照组，$Time_t\times Treat_i$ 表示干预和时间变量的交互作用。β_1 和 β_2 分别为时间变量和干预变量的系数，δ 为交互作用的系数，也正是本研究所关心的干预效果系数。ϵ_{it} 表示其他未观测到的可能影响结果指标的因素。

因此，在对应模型中加入时间变量和干预变量的交互项，以评估干预效果。针对家长 HPV 疫苗知识（连续变量），将采用混合效应模型（Mixed Effect Model）；对于家长接种咨询、HPV 疫苗信心、为女儿接种意愿（分类变量），将采用广义估计方程(Generalized estimating equation, GEE)。

4、推广阶段：通过定性访谈，评估聊天机器人的可接受性和推广性，优化聊天机器人并推广其应用范围

干预实施阶段后，我们将跟踪开展相关调查，以了解聊天机器人的可接受性和推广性。

（1）通过用户讨论，评估聊天机器人的可接受性

我们将在每个学校每个年级选择 1 名参与研究的女生家长开展个人深度访谈。具体讨论内容包括对聊天机器人的接受度、使用感受和意见，对聊天机器人服务形式和内容的偏好，对聊天机器人的期望服务内容等。

（2）通过专业人员访谈，评估聊天机器人的可推广性

我们将在每地访谈 8 名专业工作人员，来自当地疾控中心和疫苗接种点的工作人员。访谈内容包括对聊天机器人的评价、结合试验效果的改进意见以及推广应用聊天机器人的建议等。

（3）优化聊天机器人，推广其应用范围

结合上述结果，优化聊天机器人的服务内容与呈现形式；并将聊天机器人推广至对照组研究对象以及当地其他学校、接种单位，跟踪后续 HPV 疫苗聊天机器人应用状况，一段时间后选择 10 名对照组家长、8 名其他其他接种单位负责人（6 人）和工作人员（2 人）进行访谈。访谈内容同上。

5、通过实施科学研究，评估聊天机器人干预项目的适用性和可推广性

在干预效果评价和可接受性、可推广性评价基础上，本研究还将综合上述证据开展实施科学研究，采用 RE-AIM（Reach, Effectiveness, Adoption, Implementation, and Maintenance）框架来全面评价 HPV 疫苗聊天机器人干预项目在真实世界中的经济型、适用性和推广情况。采用定量与定性相结合的方法，从可及性、实施效果、采纳性、实施情况和持续性 5 个维度进行评估。见表 1。

表 1 基于 RE-AIM 框架的流感疫苗聊天机器人的实施科学评价

RE-AIM 和经济性维度	评价指标	评价时期（评价方法）
**覆盖面** (Reach)：干预项目的目标人群覆盖情况	1. 愿意参加疫苗聊天机器人干预项目的家长数量及比例；<br>2. 聊天机器人的总访问次数；	干预实施阶段（聊天机器人后台数据、问卷调查）
**有效性** (Effectiveness)：干预项目的效果	干预前后家长的 HPV 疫苗知识、信心、接种意愿及行为，见本节 3.3 调查问卷结果变量。	干预实施阶段（问卷调查、接种信息系统）
**采纳度** (Adoption)：采纳干预项目的机构和个体的数量及比例	**干预实施阶段：**<br>1. 聊天机器人可用性调查量表结果<br>2. 采纳疫苗聊天机器人作为健康教育工具的家长及疫苗接种相关机构数量及比例；<br>3. 愿意将聊天机器人推荐给别人使用的家长数量及比例<br>4. 家长和机构采纳或不采纳的原因，未来愿意接受聊天机器人的可能性和原因；	干预实施和推广阶段（问卷调查、家长和专业机构人员访谈）

**实施情况** (Implementation): 干预项目按预期内容进行实施的程度	**保真度：**<br>1. 聊天机器人访问人群中进行对话的人群占比；<br>2. 使用聊天机器人对话的平均时长及咨询问题数量；<br>**实施问题：**<br>1. 聊天机器人的内容、形式等各要素是否被接受；	干预实施阶段（聊天机器人后台数据、问卷调查、家长和机构专业人员访谈）
**可持续性** (Maintenance): 干预项目长期的持续程度	**机构层面：**疫苗聊天机器人是否成为机构和接种人员的日常工具，在疫苗接种健康教育中使用；<br>**公众层面：**<br>1. 干预结束后家长是否愿意继续使用聊天机器人，使用人数及比例的变化趋势；<br>2. 后续青少年 HPV 疫苗的接种率。	推广阶段（聊天机器人后台数据、接种信息系统）

1）实施科学评价数据来源

实施科学评价覆盖干预实施阶段与推广阶段，采用定量与定性相结合的方法。定量数据来自聊天机器人后台记录、干预阶段家长问卷调查、当地接种信息系统。定性数据来自推广阶段疾控机构、社区卫生服务中心负责人与医务人员、家长的小组讨论和深入访谈。

2）实施科学评价方法

① **实施科学定量分析：**通过描述性分析，如均值（标准差）、数量（百分比）来描述聊天机器人项目的覆盖面、采纳度、实施情况和可持续性。

② **实施科学定性分析：**利用扎根理论和主题框架分析法，总结聊天机器人覆盖和采纳的影响因素、实施障碍和问题、推广价值和建议等

6、时间安排

项目期限：2023 年 9 月至 2025 年 12 月。

2023 年 9 月-2024 年 12 月：项目完善、现场联系、基线调查、干预实施、追踪调查；

2025 年 1 月-12 月：文章撰写、研究报告撰写、研究结果传播。

7、研究现场

上海市嘉定区：震川中学、金鹤学校、苏民学校、疁城实验中学；

池州市：池州十中、十一中、十六中；

东至和青阳县：东至兰溪中学、尧城中学、青阳四中。

侯志远

复旦大学公共卫生学院

2024 年 2 月 18 日

池州市疾病预防控制中心

[illegible] 2024年2月20日

参考文献

[1] Behavioural and social drivers of vaccine uptake: tools and guidance for achieving high uptake. Geneva: World Health Organization; 2022.

[2] World Health Organization. Human papillomavirus vaccines: WHO position paper (2022 update). Weekly Epidemiological Record. 2022, 97 (50), 645-672.

[3] Hu S, Xu X, Zhang Y, et al. A nationwide post-marketing survey of knowledge, attitude and practice toward human papillomavirus vaccine in general population: Implications for vaccine roll-out. Vaccine. 2021 Jan 3;39(1):35-44.

[4] 宋祎凡,刘晓雪,尹遵栋,等.2018-2020 年中国 9-45 岁女性人乳头瘤病毒疫苗估算接种率[J].中国疫苗和免疫,2021,27(05):570-575.

[5] 邱丽蓉,牛战琴.9～14 岁女性人乳头瘤病毒疫苗接种现状及其影响因素分析[J].中国生育健康杂志,2022,33(03):262-265.

[6] Falcaro M, Castanon A, Ndlela B, et al. The effects of the national HPV vaccination programme in England, UK, on cervical cancer and grade 3 cervical intraepithelial neoplasia incidence: a register-based observational study. Lancet 2021; 398(10316): 2084-92.

[7] Drolet M, Benard E, Perez N, Brisson M, Group HPVVIS. Population-level impact and herd effects following the introduction of human papillomavirus vaccination programmes: updated systematic review and meta-analysis. Lancet 2019; 394(10197): 497-509.

[8] Sonawane K, Zhu Y, Montealegre JR, et al. Parental intent to initiate and complete the human papillomavirus vaccine series in the USA: a nationwide, cross-sectional survey. Lancet Public Health 2020; 5(9): e484-e92.

[9] Zang S, Zhang X, Qu Z, et al. Promote COVID-19 Vaccination for Older Adults in China. China CDC Wkly 2022; 4(37): 832-4.

[10] 史金晶,张肖肖,郑徽,等.中国大陆青少年家长人乳头瘤病毒疫苗认知度和接受度 Meta 分析[J].中国疫苗和免疫,2019,25(04):464-470.

[11] 武丽,马远珠,黄雪萍,等.广东省青少年女性对 HPV 疫苗知晓及接种意愿的调查研究[J].中国妇幼卫生杂志,2021,12(05):19-23.

[12] Zhang Y, Wang Y, Liu L, et al. Awareness and knowledge about human papillomavirus vaccination and its acceptance in China: a meta-analysis of 58 observational studies. BMC Public Health 2016; 16: 216.

[13] Xie Y, Su LY, Wang F, Tang HY, Yang QG, Liu YJ. Awareness regarding and vaccines acceptability of human papillomavirus among parents of middle school students in Zunyi, Southwest China. Hum Vaccin Immunother 2021; 17(11): 4406-11.

[14] Wei Z, Liu Y, Zhang L, et al. Stages of HPV Vaccine Hesitancy Among Guardians of Female Secondary School Students in China. J Adolesc Health. 2022. Epub 2022/10/14.

[15] Zhang X, Wang Z, Ren Z, et al. HPV vaccine acceptability and willingness-related factors among Chinese adolescents: a nationwide study. Hum Vaccin Immunother 2021; 17(4): 1025-32.

[16] Zhang X, Liu CR, Wang ZZ, et al. Effect of a school-based educational intervention on HPV and HPV vaccine knowledge and willingness to be vaccinated among Chinese adolescents: a multi-center intervention follow-up study. Vaccine 2020; 38(20): 3665-70.

[17] Wang D, Wu J, Du J, et al. Acceptability of and barriers to human papillomavirus vaccination in China: A systematic review of the Chinese and English scientific literature. Eur J Cancer Care (Engl) 2022; 31(3): e13566.

[18] Si M, Su X, Jiang Y, et al. Effect of an IMB Model-Based Education on the Acceptability of HPV Vaccination Among College Girls in Mainland China: A Cluster RCT. Cancer Control 2022; 29: 10732748211070719.

[19] Si M, Su X, Jiang Y, et al. An Internet-Based Education Program for Human Papillomavirus Vaccination Among Female College Students in Mainland China: Application of the Information-Motivation-Behavioral Skills Model in a Cluster Randomized Trial. J Med Internet Res. 2022 Sep 30;24(9):e37848.

[20] Luk TT, Lui JHT, Wang MP. Efficacy, Usability, and Acceptability of a Chatbot for Promoting COVID-19 Vaccination in Unvaccinated or Booster-Hesitant Young Adults: Pre-Post Pilot Study. J Med Internet Res 2022; 24(10): e39063.

[21] Kathy Leung, Kristi Lee, Saudamini Dabak et al. The effectiveness of conversational AI services on Covid-19 vaccine confidence and acceptance in Thailand, Hong Kong, and Singapore, 22 December 2022, PREPRINT (Version 1) available at Research Square [https://doi.org/10.21203/rs.3.rs-2127117/v1]

[22] Wilson L, Marasoiu M. The Development and Use of Chatbots in Public Health: Scoping Review. JMIR Hum Factors. 2022;9(4):e35882.

[23] Almalki M, Azeez F. Health Chatbots for Fighting COVID-19: a Scoping Review. Acta Inform Med 2020; 28(4): 241-7.

[24] Kobayashi T, Nishina Y, Tomoi H, et al. Corowa-kun: A messenger app chatbot delivers COVID-19 vaccine information, Japan 2021. Vaccine 2022; 40(32): 4654-62.

[25] Weeks R, Cooper L, Sangha P, et al. Chatbot-Delivered COVID-19 Vaccine Communication Message Preferences of Young Adults and Public Health Workers in Urban American Communities: Qualitative Study. J Med Internet Res 2022; 24(7): e38418.

Supplementary Document 3: Survey questionnaires

Baseline Survey Questionnaire

**I. Eligibility Assessment**	
1.	You are the girl's:<br>① Father<br>② Mother<br>③ Other relative (End of questionnaire)
2.	Has your daughter received the HPV vaccine?<br>① Already vaccinated<br>② Scheduled but not yet vaccinated<br>③ Not scheduled and not vaccinated
3.	Which type of the HPV vaccine has your daughter received or scheduled? （if Q2=1, 2）<br>① Bivalent (End of questionnaire)<br>② Quadrivalent (End of questionnaire)<br>③ Nine-valent (End of questionnaire)<br>④ Don't know/forgot (End of questionnaire)
**II. Daughter's Information**	
4.	The city where your daughter goes to school：<br>① Shanghai ② Guichi District ③ Dongzhi County ④ Qingyang County
5.	Your daughter's school:
6.	Your daughter's grade:
7.	Your daughter's class:
8.	Your daughter's age: _____ years old
9.	Is your daughter an only child? ①Yes ②No
**III. Vaccine Knowledge and Attitude**	
10.	Has your daughter received the influenza vaccine in the past two years?<br>① Yes ② No ③ Don't know
11.	Has your daughter received sex education?<br>① Yes ② No ③ Don't know
12.	How familiar are you with the HPV vaccine?<br>① Very familiar<br>② Somewhat familiar<br>③ Only know the name<br>④ Have not heard of it (Skip to Q26)
13.	Please indicate whether the following statements are true or false:

	Correct	Incorrect	I don't know
1) HPV infection is common.			

	2) HPV can be sexually transmitted.			
	3) HPV infection increases risk of cervical cancer.			
	4) The most common high-risk HPV strains are HPV-16 and 18.			
	5) HPV vaccine can effectively prevent cervical cancer and other diseases caused by HPV infection.			
	6) The optimal time to receive HPV vaccination is before sexual contact.			
	7) Cervical cancer screening is no longer needed after HPV vaccination.			
	8) HPV vaccination may lead to HPV infection.			
	9) HPV vaccination may cause infertility.			
	10) The bivalent HPV vaccine has poor efficacy.			
14.	Are you worried about your daughter being infected with HPV?<br>① Very worried<br>② Somewhat worried<br>③ Neutral<br>④ Not very worried<br>⑤ Not worried at all			
15.	How serious do you think the health risks are if a girl contracts HPV?<br>① Very serious ② Serious ③ Neutral ④ Not serious ⑤ Not serious at all			
16.	How important is the HPV vaccine for your daughter?<br>① Very important ② Important ③ Neutral ④ Not important ⑤ Not important at all			
17.	Do you believe the HPV vaccine is effective?<br>① Very effective ② Effective ③ Neutral ④ Not effective ⑤ Not effective at all			
18.	Do you believe the HPV vaccine is safe?<br>① Very safe ② Safe ③ Neutral ④ Not safe ⑤ Not safe at all			
19.	Is your daughter a priority recipient for the HPV vaccine?<br>① Yes ② No ③ I don't know			
**IV. Vaccine Information Exposure and Seeking**				
20.	In the past year, have you searched for information about the HPV vaccine using a smartphone or the internet?<br>① Yes ② No			
21.	What information did you find online? (If Q20=1)<br>①Most information recommends vaccination ②Most information recommends against vaccination ③ Mixed information without clear recommendations			
22.	Have you seen or heard negative information about the HPV vaccine?<br>① Yes ② No			
23.	What HPV vaccine-related information would you like to learn more about? *(Select all that apply)*<br><br>① Diseases prevented by the HPV vaccine<br>② Effectiveness of the HPV vaccine<br>③ Safety and side effects of the HPV vaccine			

④ Differences among bivalent, quadrivalent, and nine-valent HPV vaccines
⑤ Recommended age for HPV vaccination
⑥ Whether individuals without sexual activity need the HPV vaccine
⑦ Cost of the HPV vaccine
⑧ Prefer not to know
⑨ Others (please specify): ___________

V. Health Professional Consultation about HPV vaccine

24. In the past year, have you consulted medical professionals about HPV vaccination for your daughter?
① Yes ② No

25. What was the recommendation from medical professionals about HPV vaccination? (If Q24=1)
① Recommended to vaccinate ② Recommended against vaccination ③ No clear recommendation provided

VI. Vaccination Behavior and Intention

26. Are you willing to vaccinate your daughter against HPV?
① Strong willing ② Somewhat willing ③Neutral/Unsure ④ Not very willing ⑤Not willing at all

27. If vaccines are readily available, when would you prefer your daughter to be vaccinated? (If 26=1 or 2)
① As soon as possible
② After learning more about the vaccine
③ After consulting with medical professionals
④ After most of her classmates have been vaccinated
⑤ After she become an adult
⑥ Other (please specify): ___________

28. Which HPV vaccine do you plan for your daughter to receive? (If 26=1 or 2)
① Bivalent (2v-HPV) vaccine
② Quadrivalent (4v-HPV) vaccine
③ Nonavalent (9v-HPV) vaccine
④ No preference

29. If the nine-valent or quadrivalent vaccines are out of stock but the bivalent vaccine is available, will you wait for the preferred vaccine to be restocked? (If 28=2 or 3)
① Yes ② No

30. What are your reasons for being unsure or unwilling to vaccinate your daughter with the HPV vaccine? (If 26=3, 4, or 5)

		Yes	No
1)	The HPV vaccine has poor efficacy.		
2)	The HPV vaccine is not very safe.		
3)	My daughter will not get HPV infection.		
4)	HPV infection is not serious.		
5)	I lack sufficient knowledge to decide.		
6)	My family and friends don’t recommend the HPV vaccine.		
7)	The HPV vaccine is expensive.		

31. Have you been vaccinated with the HPV vaccine? (If Q1=2)

	① Yes ② No
32.	Has your spouse received the HPV vaccine? (If Q1=1)<br>① Yes ② No ③ Don’t know
**VII. Family Information**	
33.	Your age: __ years old
34.	Your level of education:<br>① Primary school or below<br>② Junior high school<br>③ High school/vocational school<br>④ College/Bachelor's degree<br>⑤ Master's degree or above
35.	Your occupation?<br>① Medical professional<br>② Government or public institution employee<br>③ Biopharmaceutical company employee<br>④ Other company employee<br>⑤ Freelancer<br>⑥ Worker<br>⑦ Farmer<br>⑧ Self-employed<br>⑨ Other
36.	Your family's total annual income:<br>① Below 100,000 RMB<br>② 100,000-200,000 RMB<br>③ 200,000-300,000 RMB<br>④ 300,000-500,000 RMB<br>⑤ Above 500,000 RMB
37.	In the past six months, has your daughter lived together with her parents?<br>① Lived together with both parents<br>② Lived only with mother<br>③ Lived only with father<br>④ Not living with either parent
38.	If not living together, is it because you or your spouse went to work elsewhere? (If Q37=2, 3 or 4)<br>① Out of province<br>② Within the province but different city<br>③ Within the same city
39.	In which province are you or your spouse working? (If Q38=1）<br>① Shanghai, Beijing, Guangzhou, Shenzhen<br>② Zhejiang<br>③ Jiangsu<br>④ Other areas in Guangdong (excluding Guangzhou and Shenzhen)<br>⑤ Other provinces
40.	The last four digits of your phone number: _____

Follow-up Survey Questionnaire

**I. Eligibility Assessment**	
41.	You are the girl's:<br>④ Father<br>⑤ Mother<br>⑥ Other relative (End of questionnaire)
42.	Did you complete the “Middle School Parents' HPV Vaccine Questionnaire” two weeks ago?<br>① Yes ② No (End of questionnaire)
43.	Before filling out the baseline questionnaire, had your daughter already received or scheduled the HPV vaccine?<br>① Yes (End of questionnaire) ② No
**II. Daughter's Information**	
44.	The city where your daughter goes to school：<br>① Shanghai ② Guichi District ③ Dongzhi County ④ Qingyang County
45.	Your daughter's school:
46.	Your daughter's grade:
47.	Your daughter's class:
**III. HPV Vaccine Knowledge and Attitude**	
48.	How familiar are you with the HPV vaccine?<br>① Very familiar<br>② Somewhat familiar<br>③ Only know the name<br>④ Have not heard of it (Skip to Q18)
49.	Please indicate whether the following statements are true or false:

	Correct	Incorrect	I don’t know
11) HPV infection is common.			
12) HPV can be sexually transmitted.			
13) HPV infection increases risk of cervical cancer.			
14) The most common high-risk HPV strains are HPV-16 and 18.			
15) HPV vaccine can effectively prevent cervical cancer and other diseases caused by HPV infection.			
16) The optimal time to receive HPV vaccination is before sexual contact.			
17) Cervical cancer screening is no longer needed after HPV vaccination.			
18) HPV vaccination may lead to HPV infection.			
19) HPV vaccination may cause infertility.			
20) The bivalent HPV vaccine has poor efficacy.			

50.	Are you worried about your daughter being infected with HPV?

	⑦ Very worried<br>⑧ Somewhat worried<br>⑨ Neutral<br>⑩ Not very worried<br>⑪ Not worried at all
51.	How serious do you think the health risks are if a girl contracts HPV?<br>① Very serious ② Serious ③ Neutral ④ Not serious ⑤ Not serious at all
52.	How important is the HPV vaccine for your daughter?<br>① Very important ② Important ③ Neutral ④ Not important ⑤ Not important at all
53.	Do you believe the HPV vaccine is effective?<br>① Very effective ② Effective ③ Neutral ④ Not effective ⑤ Not effective at all
54.	Do you believe the HPV vaccine is safe?<br>① Very safe ② Safe ③ Neutral ④ Not safe ⑤ Not safe at all
55.	Is your daughter a priority recipient for the HPV vaccine?<br>① Yes ② No ③ I don't know
**IV. Vaccine Information Exposure and Seeking**	
56.	In the past two weeks (since the baseline questionnaire), have you searched for HPV vaccine-related information via mobile phone or online?<br>① Yes ② No
**V. Health Professional Consultation about HPV vaccine**	
57.	In the past two weeks (since the baseline questionnaire), have you consulted medical professionals about vaccinating your daughter with the HPV vaccine?<br>① Yes ② No
**VI. Vaccination Behavior and Intention**	
58.	In the past two weeks (since the baseline questionnaire), has your daughter been vaccinated or scheduled to be vaccinated with the HPV vaccine?<br>① Vaccinated ② Scheduled but not yet vaccinated ③ Not scheduled and not vaccinated
59.	Which HPV vaccine did she receive or schedule? (If 18= 1 or 2)<br>① Bivalent (2v-HPV) vaccine<br>② Quadrivalent (4v-HPV) vaccine<br>③ Nonavalent (9v-HPV) vaccine
60.	Are you willing to vaccinate your daughter against HPV? (If 18=3)<br>① Strongly willing ② Somewhat willing ③ Neutral/Unsure ④ Not very willing ⑤ Not willing at all
61.	If vaccines are readily available, when would you prefer your daughter to be vaccinated? (If 20=1 or 2)<br>① As soon as possible<br>② After learning more about the vaccine<br>③ After consulting with medical professionals<br>④ After most of her classmates have been vaccinated<br>⑤ After she become an adult<br>⑥ Other time (Please specify)
62.	Which HPV vaccine do you plan for your daughter to receive? (If 20=1 or 2)<br>① Bivalent (2v-HPV) vaccine

	② Quadrivalent (4v-HPV) vaccine<br>③ Nonavalent (9v-HPV) vaccine<br>④ No preference
63.	If the nine-valent or quadrivalent vaccines are out of stock but the bivalent vaccine is available, will you wait for the preferred vaccine to be restocked? (If 22=2 or 3)<br>① Yes ② No
64.	What are your reasons for being unsure or unwilling to vaccinate your daughter with the HPV vaccine? (If 20=3, 4, or 5)

		Yes	No
8)	The HPV vaccine has poor efficacy.		
9)	The HPV vaccine is not very safe.		
10)	My daughter will not get HPV infection.		
11)	HPV infection is not serious.		
12)	I lack sufficient knowledge to decide.		
13)	My family and friends don't recommend the HPV vaccine.		
14)	The HPV vaccine is expensive.		

**VII. Family Information**	
65.	Your age: __ years old
66.	The last four digits of your phone number: ____

**VIII. Chatbot Usage Experience Assessment (Intervention Group Only)**	
67.	In the past two weeks (since the baseline questionnaire), have you used an HPV vaccine chatbot?<br>① Yes<br>② No (skip the following questions)
68.	How many times have you used it in total?
69.	Total duration of use: __ minutes
70.	How many questions about the HPV vaccine did you ask?
71.	After using the HPV vaccine chatbot, to what extent do you agree with the following statements?

	Strongly agree	Somewhat agree	Neutral/Don't know	Somewhat disagree	Strongly disagree
1) I believe the chatbot has helped me better understand the effectiveness and uses of the HPV vaccine.					
2) I believe the chatbot always understands what I express and provides appropriate responses.					
3) I believe the chatbot is user-friendly and easy to use.					
4) I believe the chatbot has					

		enabled me to obtain HPV vaccine-related information more efficiently.					
		5) I believe the HPV vaccine information provided by the chatbot is accurate and reliable.					
		6) I believe the HPV vaccine information provided by the chatbot is clearly expressed and easy to understand.					
		7) I believe the chatbot is impartial and unbiased when providing HPV vaccine information.					
		8) I believe it is safe to use the chatbot to inquire about HPV vaccine information.					
		9) I believe the chatbot adheres to ethical standards when providing HPV vaccine information.					
		10) I believe the chatbot can properly protect my personal privacy when handling my inquiry data.					
		11) When answering HPV vaccine questions, I believe the chatbot is comparable to, or even better than, professionals.					
		12) I am willing to use the chatbot to obtain information about the HPV vaccine.					
		13) I have certain concerns about using the chatbot to consult HPV vaccine information.					
		14) I am very clear about the chatbot's functions and its					

		ability to process HPV vaccine information.					
		15) Overall, I am satisfied with the performance of the chatbot.					
		16) I would use the chatbot again if I had the chance.					
		17) I would recommend the chatbot to others if I had the chance.					

CONSORT 2010 checklist of information to include when reporting a randomised trial*

Section/Topic	Item No	Checklist item	Reported on page No
**Title and abstract**			
	1a	Identification as a randomised trial in the title	1
	1b	Structured summary of trial design, methods, results, and conclusions (for specific guidance see CONSORT for abstracts)	1-2
**Introduction**			
Background and objectives	2a	Scientific background and explanation of rationale	2-3
	2b	Specific objectives or hypotheses	3
**Methods**			
Trial design	3a	Description of trial design (such as parallel, factorial) including allocation ratio	17
	3b	Important changes to methods after trial commencement (such as eligibility criteria), with reasons	NA
Participants	4a	Eligibility criteria for participants	17
	4b	Settings and locations where the data were collected	17
Interventions	5	The interventions for each group with sufficient details to allow replication, including how and when they were actually administered	17-19
Outcomes	6a	Completely defined pre-specified primary and secondary outcome measures, including how and when they were assessed	19-20
	6b	Any changes to trial outcomes after the trial commenced, with reasons	NA
Sample size	7a	How sample size was determined	20-21
	7b	When applicable, explanation of any interim analyses and stopping guidelines	NA
Randomisation:			
Sequence generation	8a	Method used to generate the random allocation sequence	17
	8b	Type of randomisation; details of any restriction (such as blocking and block size)	17
Allocation concealment mechanism	9	Mechanism used to implement the random allocation sequence (such as sequentially numbered containers), describing any steps taken to conceal the sequence until interventions were assigned	17
Implementation	10	Who generated the random allocation sequence, who enrolled participants, and who assigned participants to interventions	17
Blinding	11a	If done, who was blinded after assignment to interventions (for example, participants, care providers, those	17

		assessing outcomes) and how	
	11b	If relevant, description of the similarity of interventions	NA
Statistical methods	12a	Statistical methods used to compare groups for primary and secondary outcomes	21
	12b	Methods for additional analyses, such as subgroup analyses and adjusted analyses	21
**Results**			
Participant flow (a diagram is strongly recommended)	13a	For each group, the numbers of participants who were randomly assigned, received intended treatment, and were analysed for the primary outcome	3
	13b	For each group, losses and exclusions after randomisation, together with reasons	3
Recruitment	14a	Dates defining the periods of recruitment and follow-up	3
	14b	Why the trial ended or was stopped	NA
Baseline data	15	A table showing baseline demographic and clinical characteristics for each group	9-10
Numbers analysed	16	For each group, number of participants (denominator) included in each analysis and whether the analysis was by original assigned groups	3
Outcomes and estimation	17a	For each primary and secondary outcome, results for each group, and the estimated effect size and its precision (such as 95% confidence interval)	4
	17b	For binary outcomes, presentation of both absolute and relative effect sizes is recommended	4
Ancillary analyses	18	Results of any other analyses performed, including subgroup analyses and adjusted analyses, distinguishing pre-specified from exploratory	4-6
Harms	19	All important harms or unintended effects in each group (for specific guidance see CONSORT for harms)	NA
**Discussion**			
Limitations	20	Trial limitations, addressing sources of potential bias, imprecision, and, if relevant, multiplicity of analyses	7
Generalisability	21	Generalisability (external validity, applicability) of the trial findings	7
Interpretation	22	Interpretation consistent with results, balancing benefits and harms, and considering other relevant evidence	6-7
**Other information**			
Registration	23	Registration number and name of trial registry	1
Protocol	24	Where the full trial protocol can be accessed, if available	Supplementary
Funding	25	Sources of funding and other support (such as supply of drugs), role of funders	8

Citation: Schulz KF, Altman DG, Moher D, for the CONSORT Group. CONSORT 2010 Statement: updated guidelines for reporting parallel group randomised trials. BMC Medicine. 2010;8:18.
© 2010 Schulz et al. This is an Open Access article distributed under the terms of the Creative Commons Attribution License (http://creativecommons.org/licenses/by/2.0), which permits unrestricted use, distribution, and reproduction in any medium, provided the original work is properly cited.

*We strongly recommend reading this statement in conjunction with the CONSORT 2010 Explanation and Elaboration for important clarifications on all the items. If relevant, we also recommend reading CONSORT extensions for cluster randomised trials, non-inferiority and equivalence trials, non-pharmacological treatments, herbal interventions, and pragmatic trials. Additional extensions are forthcoming: for those and for up-to-date references relevant to this checklist, see www.consort-statement.org.
