# Supplementary material for: A vaccine chatbot intervention for parents to improve HPV vaccination uptake among middle school girls: a cluster randomized trial
Source: Nat Med. 2025 Apr 7;31(6):1855–62. doi: 10.1038/s41591-025-03618-6 (PMC12176647; doi:10.1038/s41591-025-03618-6)
Supplement: Supplementary file 2 — Reporting Summary [file 41591_2025_3618_MOESM2_ESM.pdf]

nature portfolio

Corresponding author(s): Dr. Leesa Lin, Dr. Zhiyuan Hou

Last updated by author(s): Feb 19, 2025

Reporting Summary

Nature Portfolio wishes to improve the reproducibility of the work that we publish. This form provides structure for consistency and transparency in reporting. For further information on Nature Portfolio policies, see our Editorial Policies and the Editorial Policy Checklist.

Statistics

For all statistical analyses, confirm that the following items are present in the figure legend, table legend, main text, or Methods section.

n/a	Confirmed	
☐	☒	The exact sample size (*n*) for each experimental group/condition, given as a discrete number and unit of measurement
☐	☒	A statement on whether measurements were taken from distinct samples or whether the same sample was measured repeatedly
☐	☒	The statistical test(s) used AND whether they are one- or two-sided<br>*Only common tests should be described solely by name; describe more complex techniques in the Methods section.*
☐	☒	A description of all covariates tested
☐	☒	A description of any assumptions or corrections, such as tests of normality and adjustment for multiple comparisons
☐	☒	A full description of the statistical parameters including central tendency (e.g. means) or other basic estimates (e.g. regression coefficient) AND variation (e.g. standard deviation) or associated estimates of uncertainty (e.g. confidence intervals)
☐	☒	For null hypothesis testing, the test statistic (e.g. *F*, *t*, *r*) with confidence intervals, effect sizes, degrees of freedom and *P* value noted<br>*Give P values as exact values whenever suitable.*
☒	☐	For Bayesian analysis, information on the choice of priors and Markov chain Monte Carlo settings
☒	☐	For hierarchical and complex designs, identification of the appropriate level for tests and full reporting of outcomes
☒	☐	Estimates of effect sizes (e.g. Cohen's *d*, Pearson's *r*), indicating how they were calculated

Our web collection on statistics for biologists contains articles on many of the points above.

Software and code

Policy information about availability of computer code

Data collection	Data were collected through a self-administered questionnaire hosted on the Wenjuanxing online survey platform.
Data analysis	Statistical analyses were performed using STATA 15.1 and R version 4.4.1. All codes are available on GitHub at https://github.com/wu-zhengdong/HPV-vaccine-chatbot.git.

For manuscripts utilizing custom algorithms or software that are central to the research but not yet described in published literature, software must be made available to editors and reviewers. We strongly encourage code deposition in a community repository (e.g. GitHub). See the Nature Portfolio guidelines for submitting code & software for further information.

Data

Policy information about availability of data

All manuscripts must include a data availability statement. This statement should provide the following information, where applicable:

- Accession codes, unique identifiers, or web links for publicly available datasets
- A description of any restrictions on data availability
- For clinical datasets or third party data, please ensure that the statement adheres to our policy

The data are not publicly available, and making the data publicly available would require additional consent due to the need to protect participant privacy and confidentiality. Researchers interested in accessing the data should submit requests to the corresponding author (Dr. Leesa Lin, leesa.lin@lshtm.ac.uk or Dr. Zhiyuan Hou, zyhou@fudan.edu.cn), explaining the analyses planned. Access to data will be provided upon application, with a timeline of one month determined in

accordance with the request. All approved users must sign a data use agreement that specifies confidentiality requirements, restricts data usage to the approved analyses, and prohibits any attempt to identify study participants.

Research involving human participants, their data, or biological material

Policy information about studies with human participants or human data. See also policy information about sex, gender (identity/presentation), and sexual orientation and race, ethnicity and racism.

Reporting on sex and gender	This study enrolled female students (biological sex) and their parents. Parental roles (father or mother) were self-identified, with gender inferred from these roles (father as male, mother as female).
Reporting on race, ethnicity, or other socially relevant groupings	Race and ethnicity were not specifically analyzed in this study. The participant population was diverse in terms of socioeconomic distribution across different regions in China, including the metropolitan area of Shanghai Jiading, the urban setting of Guichi in Anhui Province, and the rural counties of Dongzhi and Qingyang, also in Anhui Province.
Population characteristics	Participants were parents (mean age: 40.4 ± 4.6 years) of female middle school students (grades 6-9, mean age: 13.1 ± 1.1 years). Study examined parents' characteristics including age, education level (43.7% high education, 18.0% senior high school, 38.3% junior middle school and below), employment status (30.9% formally employed), annual household income (9.7% >300k CNY, 11.8% 200-300k CNY, 34.4% 100-200k CNY, 44.1% <100k CNY), and HPV vaccination status (23.7% vaccinated). The majority (87.7%) were mothers. Daughters' characteristics included age, only-child status (38.6%), left-behind status (5.1% living with grandparents or guardians), sexual education exposure (59.5%), and influenza vaccination history (30.5% vaccinated within 2 years). Groups were balanced to ensure comparability across all measured characteristics.
Recruitment	Participants were recruited from 180 classes across 10 middle schools (3-4 schools per region) in three regions (megacity, urban, and rural), with schools selected based on economic development and geographical location. Approximately 20 parents per class were invited through school communication channels (parent-teacher meetings and school announcements). Eligible participants were parents of female students (grades 6-9) whose children had not received HPV vaccination, had no contraindications, and who could fully participate in study activities with informed consent. Potential biases include: the targeted selection of schools limiting generalizability; survey completion by different parents within families affecting data reliability; possible cross-contamination between intervention and control classes within schools; and connectivity issues in rural areas. Factors that may underestimate intervention effects include the required out-of-pocket payment, vaccine supply shortages, and baseline survey completion in the control group increasing vaccine interest.
Ethics oversight	Ethical oversight was provided by the IRB of Fudan University and the HREC of the University of Hong Kong. Informed consent was obtained from all participants.

Note that full information on the approval of the study protocol must also be provided in the manuscript.

Field-specific reporting

Please select the one below that is the best fit for your research. If you are not sure, read the appropriate sections before making your selection.

☐ Life sciences ☒ Behavioural & social sciences ☐ Ecological, evolutionary & environmental sciences

For a reference copy of the document with all sections, see nature.com/documents/nr-reporting-summary-flat.pdf

Behavioural & social sciences study design

All studies must disclose on these points even when the disclosure is negative.

Study description	This was a two-arm cluster randomized trial (cRCT) with quantitative data analysis.
Research sample	A total of 180 classes were selected from 10 junior middle schools in mainland China (Shanghai metropolitan area and Anhui province's urban and rural regions). These regions were selected as they required out-of-pocket payment for HPV vaccination without government subsidies. After exclusions, 2,671 parents were analyzed: 1,294 in the intervention group and 1,377 in the control group. The sample comprised primarily mothers (87.7%, mean age 40.4±4.6 years) of female students (mean age 13.1±1.1 years, grades 6-9), representing diverse socioeconomic backgrounds and geographical settings. The sample size was calculated based on expected vaccination rates, cluster design effect, and anticipated attrition. While enrolled participants were comparable to non-enrolled parents in most characteristics, the findings may be limited in generalizability to regions with different vaccination policies.
Sampling strategy	This study employed a stratified cluster random sampling strategy in a cluster randomized clinical trial. From three regions in mainland China (Shanghai metropolitan area, urban Guichi, and rural Dongzhi and Qingyang in Anhui Province), 10 middle schools were selected based on economic development and geographical location. The sample size was calculated based on detecting an increase in HPV vaccination uptake among middle school girls from 5% to 10%, with 80% power and a significance level of 0.05, incorporating intracluster correlation coefficient as 0.05 and a cluster design effect of 1.5 (with an average of 11 students per cluster). This calculation determined that 648 participants per arm would be required. Accounting for an anticipated 20-30% loss to follow-up, the sample size was expanded to 900 participants per arm (total n=1,800). From the selected schools, 180 classes (grades 6-9) were identified, and classes as clusters were randomly assigned to intervention or control groups in a 1:1 ratio using computer-generated randomization, stratified by region, school, and grade level. One parent of each eligible female student (aged 12-15 years) from the selected classes was invited to participate if their child had not received HPV vaccination, had no scheduled vaccination

	appointment, and had no contraindications to vaccination.
Data collection	Two rounds of survey were conducted before and after the two-week intervention period. Data were collected through an online survey platform (Wenjuanxing), with class teachers distributing printed letters containing survey QR codes to students for their parents and promoting the study through digital parent communities. Both survey questionnaires were provided in the Supplementary Documents. All participants were provided informed consent during their screening/baseline visits and completed the survey independently. HPV vaccination was verified through official vaccination records during the follow-up assessment. The nature of the intervention did not allow for masking of the intervention to class teachers, participants or study researchers.
Timing	The intervention was conducted over a period of 2 weeks within the overall trial timeline from January 18, 2024, to May 31, 2024.
Data exclusions	Participants were enrolled if they met the following inclusion criteria: (1) participants were parents of female students currently enrolled in participating middle schools (grades 6-9, where students typically range from 12-15 years of age); (2) the female HPV-vaccine eligible child of the surveyed parent had not received an HPV vaccine, did not have an HPV vaccination appointment scheduled, and did not have any contraindications to receiving the HPV vaccine; (3) participants were free of mental health disorders or visual/reading disabilities that could prevent their full participation in and completion of the intervention activities; and (4) participants provided informed consent and expressed a willingness to actively participate throughout the study. Exclusion criteria were defined as individuals not meeting the aforementioned inclusion criteria. During the initial screening phase, a total of 590 parents were excluded from the study (235 parents whose children had already received or were scheduled to receive HPV vaccine, 346 parents due to non-response, and 9 parents due to invalid response).
Non-participation	A total of 633 participants were lost to follow-up during the study.
Randomization	Classes were randomly assigned to intervention or control group (1:1 ratio) using stratified randomization by region (megacity, urban, rural), school (n=10), and grade (6-9). Within each grade, computer-generated randomization determined whether Class 1 and 2 were assigned to intervention or control group, with odd-numbered classes following Class 1's assignment and even-numbered classes following Class 2's. Randomization was blinded to schools, teachers, and participants, though intervention masking was not possible.

Reporting for specific materials, systems and methods

We require information from authors about some types of materials, experimental systems and methods used in many studies. Here, indicate whether each material, system or method listed is relevant to your study. If you are not sure if a list item applies to your research, read the appropriate section before selecting a response.

Materials & experimental systems

n/a	Involved in the study	
☒	☐	Antibodies
☒	☐	Eukaryotic cell lines
☒	☐	Palaeontology and archaeology
☒	☐	Animals and other organisms
☐	☒	Clinical data
☒	☐	Dual use research of concern
☒	☐	Plants

Methods

n/a	Involved in the study	
☒	☐	ChIP-seq
☒	☐	Flow cytometry
☒	☐	MRI-based neuroimaging

Clinical data

Policy information about clinical studies

All manuscripts should comply with the ICMJE guidelines for publication of clinical research and a completed CONSORT checklist must be included with all submissions.

Clinical trial registration	This trial was registered with ClinicalTrials.gov (identifier: NCT06227689).
Study protocol	The study protocol is unpublished but has been submitted as a supplementary file.
Data collection	Data were collected from three representative regions in China, including the metropolitan area of Shanghai Jiading, the urban setting of Guichi in Anhui Province, and the rural counties of Dongzhi and Qingyang in Anhui Province, within the overall trial timeline from January 18, 2024, to May 31, 2024.
Outcomes	The primary outcome was the receipt or scheduled appointment of the HPV vaccine, determined by whether the participants' daughters were vaccinated or had actively scheduled a vaccination within the two-week intervention period. Secondary outcomes included: (1) whether participants had consulted health professionals about getting their daughters vaccinated against HPV within the two-week post-intervention period; (2) parental willingness to vaccinate their daughters against HPV; (3) HPV vaccination literacy and (4) HPV vaccine confidence.

Plants

Seed stocks

Report on the source of all seed stocks or other plant material used. If applicable, state the seed stock centre and catalogue number. If plant specimens were collected from the field, describe the collection location, date and sampling procedures.

Novel plant genotypes

Describe the methods by which all novel plant genotypes were produced. This includes those generated by transgenic approaches, gene editing, chemical/radiation-based mutagenesis and hybridization. For transgenic lines, describe the transformation method, the number of independent lines analyzed and the generation upon which experiments were performed. For gene-edited lines, describe the editor used, the endogenous sequence targeted for editing, the targeting guide RNA sequence (if applicable) and how the editor was applied.

Authentication

Describe any authentication procedures for each seed stock used or novel genotype generated. Describe any experiments used to assess the effect of a mutation and, where applicable, how potential secondary effects (e.g. second site T-DNA insertions, mosiacism, off-target gene editing) were examined.
